# Soluble triggering receptor expressed on myeloid cells 1 (sTREM-1) predicts mortality in patients with febrile illness in southern Mozambique

**DOI:** 10.1038/s43856-025-01014-2

**Published:** 2025-07-25

**Authors:** Núria Balanza, Bàrbara Baro, Sara Ajanovic, Zumilda Boca, Justina Bramugy, Anelsio Cossa, Elizabeth JA. Fitchett, Heidi Hopkins, Suzanne H. Keddie, Sham Lal, David C. W. Mabey, Tegwen Marlais, Hridesh Mishra, Campos Mucasse, Marta Valente, Andrea M. Weckman, Julie K. Wright, Shunmay Yeung, Kathleen Zhong, Kevin C. Kain, Quique Bassat

**Affiliations:** 1 ISGlobal, Barcelona, Spain; 2 Facultat de Medicina i Ciències de la Salut, Universitat de Barcelona (UB), Barcelona, Spain; 3 Centro de Investigação em Saúde de Manhiça (CISM), Maputo, Mozambique; 4 London School of Hygiene and Tropical Medicine, London, UK; 5 Sandra-Rotman Centre for Global Health, Toronto General Research Institute, University Health Network-Toronto General Hospital, Toronto, ON Canada; 6 Tropical Disease Unit, Division of Infectious Diseases, Department of Medicine, University of Toronto, Toronto, ON Canada; 7 Department of Laboratory Medicine and Pathobiology, University of Toronto, Toronto, ON Canada; 8 ICREA, Barcelona, Spain; 9 Institut Clínic de Medicina i Dermatologia, Hospital Clínic de Barcelona, Barcelona, Spain; 10 Paediatrics Department, Hospital Sant Joan de Déu—University of Barcelona, Esplugues, Barcelona, Spain; 11 CIBER de Epidemiología y Salud Pública, Instituto de Salud Carlos III, Madrid, Spain

**Keywords:** Prognostic markers, Infectious diseases, Prognostic markers, Prognosis

## Abstract

**Background:**

Fever is a leading reason for seeking healthcare globally. Early in the course of febrile illness, it is challenging to identify patients at risk of severe and fatal infections. Quantifying biomarkers of immune and endothelial activation may facilitate patient triage.

**Methods:**

We prospectively enrolled children ≥2 months and adults with fever visiting two Mozambican hospitals from December 2018 to February 2021. Standard clinical and laboratory parameters, including lactate levels, were assessed at presentation. Plasma levels of Angpt-2, CHI3L1, CRP, IL-6, IL-8, PCT, sFlt-1, sTNFR1, sTREM-1, and suPAR at presentation were retrospectively quantified. Clinical outcomes were evaluated up to 28 days. We assessed the prognostic performance of biomarkers for 28-day mortality and explored their association with other adverse outcomes.

**Results:**

This study includes 1955 participants, with 93 deaths occurring within 28 days. We show that all biomarker levels are elevated in inpatients compared to outpatients and are associated with 28-day mortality (all p < 0.001). sTREM-1 is the best biomarker predicting 28-day mortality with an AUROC of 0.82 (95% CI: 0.78-0.86), superior to that of PCT (p < 0.001), CRP (p < 0.001), and lactate (p = 0.0033). Its prognostic performance is consistent across age and sex, but is reduced in HIV-positive individuals (AUROC = 0.73, 95% CI: 0.66-0.80). Adding sTREM-1 improves the discrimination of clinical severity scores for 28-day mortality. Among discharged inpatients, sTREM-1 is positively correlated with duration of hospitalisation (p < 0.001). Among outpatients, sTREM-1 levels are higher in those seeking further care (p = 0.0022) or subsequently hospitalised (p = 0.012).

**Conclusions:**

sTREM-1 is a promising biomarker for risk stratification of all-age, all-cause febrile illnesses in resource-limited settings.

## Plain language summary

Fever is a common reason people seek healthcare, but at presentation it is often difficult to determine which individuals are at risk of becoming seriously ill. This study followed children and adults with fever presenting to hospital in Mozambique to assess whether measuring certain blood biomarkers could help identify those at risk of severe and fatal infections. One biomarker, called sTREM-1, was the best predictor of mortality and added prognostic information beyond usual clinical signs in both children and adults. sTREM-1 was slightly less accurate in HIV-positive patients but remained a good predictor. High levels of sTREM-1 were also linked to other adverse outcomes besides mortality. Measuring sTREM-1 could help healthcare workers in resource-limited settings recognize febrile patients who require closer monitoring or advanced treatment.

## Abstract (web summary)

Balanza et al. study biomarkers of immune and endothelial activation in children and adults with febrile illness presenting to hospital in Mozambique. They find that sTREM-1 predicts 28-day mortality and represents a promising biomarker for patient risk stratification.

## Introduction

Febrile illnesses are a leading reason for clinical consultations globally, especially in low- and middle-income countries where the prevalence of infections is higher. Although fever can result from non-infectious causes, infections are a common trigger^1^. It is estimated that ~80% of individuals attending primary care facilities across Africa for an acute medical condition had self-reported or documented fever^1^. At the primary care level, many febrile illnesses are uncomplicated and self-resolving. Only a small proportion involves a life-threatening infection requiring prompt and intensive intervention, including hospitalisation or referral for higher-level care^2^. However, early in the course of febrile illness, it is difficult to identify individuals at risk of severe and fatal infections among the majority who are not, since initial presentations of life-threatening infections may be subtle and non-specific^2^. In resource-limited settings, management of febrile illnesses is further complicated by the chronic shortage of clinicians and limited laboratory services and equipment^3^. Clinical guidelines based on symptoms and signs are commonly used to diagnose and treat common infections, with the triage of clinical presentations based on a subset of danger signs that involve subjective recognition and lack sensitivity and specificity for adverse outcomes. It is therefore challenging to prioritize limited resources to those at highest risk of poor prognosis, and failure to identify severe disease results in unsafe discharge and increased mortality or long-term sequelae^4–7^.

Severe infections share common pathophysiological pathways. Endothelial and immune activation can contribute to endothelial destabilisation and organ dysfunction, being key determinants of progression to sepsis irrespective of aetiology. A growing body of evidence supports the hypothesis that measuring mediators of these pathways early in the disease course may help to improve risk stratification and management of febrile illnesses^8,9^. Biomarkers have the advantage of being quantitative, objective indicators suitable for incorporation into potentially simple and inexpensive point-of-care rapid tests^9^. Research conducted both in children and adults has shown that endothelial and immune activation biomarkers are promising prognostic tools across multiple infection aetiologies, including studies in sub-Saharan populations^10–16^. However, most studies have been performed in patients admitted to hospital with a specific aetiology or syndrome. There is limited evidence available on the relationship between biomarker levels and adverse outcomes from patients of all ages, for all-cause fevers, and in non-hospitalised individuals. Moreover, it remains unclear whether the prognostic performance of biomarkers differs according to individual patient characteristics.

In this study, we evaluate the prognostic performance of immune and endothelial activation biomarkers at clinical presentation for 28-day mortality in a large prospective cohort of febrile paediatric and adult patients presenting to hospital in Mozambique, who were managed as outpatients or inpatients. We also examine potential differences in biomarker prognostic performance across patient subgroups, their performance in relation to clinical severity scores, and their association with other adverse clinical outcomes besides mortality. Among the different biomarkers we evaluate, soluble triggering receptor expressed on myeloid cells 1 (sTREM-1) emerges as the best predictor of 28-day mortality in our study cohort and represents a promising biomarker for risk stratification of all-age, all-cause febrile illnesses in resource-limited settings.

## Methods

### Study design and participants

This is a prospective cohort study. It was conducted in the Mozambican patient cohort of the Febrile Illness Evaluation in a Broad Range of Endemicities (FIEBRE) study, a multisite prospective observational study designed to identify the infectious causes of fever in Africa and Asia^17^. Enrolment of participants in Mozambique was performed between December 2018 and February 2021. Individuals who sought care at Manhiça District Hospital (Manhiça, Mozambique) or General José Macamo Hospital (Maputo, Mozambique) were clinically evaluated. Decisions regarding whether patients were admitted to hospital or discharged home were made by non-study clinicians according to routine practice. Shortly after this decision, patients were assessed for study recruitment by trained clinical study staff and enrolled if they fulfilled the selection criteria. Enrolment was stratified and aimed to achieve balanced samples of 600 child outpatients, 600 child inpatients, 600 adult outpatients, and 600 adult inpatients. This approach was in accordance with the sample size considerations made for the primary objective of the FIEBRE study. Children were defined as participants aged ≥2 months to <15 years. In General José Macamo Hospital, only child and adult inpatients were recruited. Enrolled participants were aged ≥2 months, had tympanic or axillary temperature ≥37.5 °C at presentation, had not been hospitalised or undergone surgery in the preceding month, and were willing and able to provide demographic and clinical information and samples at enrolment and 28 days later. Outpatient selection criteria also included residence within the Manhiça district. All outpatients with symptoms of diarrhoeal diseases ( ≥ 3 loose stools within the preceding 24 h) and adult outpatients with symptoms of lower respiratory infection (cough productive of green/yellow sputum or haemoptysis) were excluded. This was for reasons related to the primary objective of the FIEBRE study, supported by pre-existing published evidence on the causes of pneumonia and diarrhoea in Africa and Asia^18,19^.

### Study procedures

Upon enrolment, participants had a complete clinical history and physical exam systematically recorded. Venous blood and other clinical samples were taken for patient care and research purposes. Details on the methods and the standard operating procedures of the FIEBRE study are available from the published study protocol^17^. Besides specific diagnostic tests for determination of cause of fever, the standard care provided by health facility staff was unchanged. Human immunodeficiency virus (HIV) status was considered positive if self-reported by the participant/caregiver or confirmed through point-of-care testing (using antibody-detecting rapid tests) performed for all participants with unknown or negative self-reported HIV status. Lactate, considered a severity marker, was measured at clinical presentation on-site using venous blood and a point-of-care analyser (Lactate Scout, SensLab GmbH). Additional clinical data were collected for inpatients during admission and at discharge. A follow-up visit was set at day 28 after enrolment (acceptable range of 26-48 days, inclusive), during which vital status and clinical data related to the original illness episode were documented. Data on seeking further care and subsequent hospitalisation in outpatients were self-reported by participants or reported by parents/guardians. All data were collected using electronic case report forms in Open Data Kit (ODK)^20^.

### Biomarker quantification

Host biomarkers with demonstrated prognostic value in acute infections were chosen for assessment, based on our prior research and review of the literature^10,11,16^. C-reactive protein (CRP) and procalcitonin (PCT) were included as predicate biomarkers commonly used in high-income settings. The ten selected biomarkers were angiopoietin-2 (Angpt-2), chitinase-3-like protein 1 (CHI3L1), CRP, interleukin-6 (IL-6), interleukin-8 (IL-8), PCT, soluble fms-like tyrosine kinase-1 (sFlt-1), soluble tumour necrosis factor receptor 1 (sTNFR1), sTREM-1, and soluble urokinase-type plasminogen activator receptor (suPAR).

Plasma obtained from EDTA-anticoagulated venous blood was obtained for all study participants at clinical presentation and stored at −80 °C in Manhiça, Mozambique. After study completion, samples were shipped to University Health Network - Toronto General Hospital in Toronto, Canada, for biomarker measurement. Samples were stored at −80 °C without freeze-thaw until batch analyte quantification. A single plasma sample from each participant was used to measure all biomarkers. Plasma concentrations of Angpt-2, CHI3L1, IL-6, IL-8, PCT, sFlt-1, sTNFR1, and sTREM-1 were quantified using the multiplex Luminex platform with custom-developed reagents from R&D Systems. Individual Luminex plates included 10% duplicates. CRP was quantified by DuoSet® enzyme-linked immunosorbent assay (ELISA) kits (R&D Systems) and all assays were run in duplicate. suPAR was quantified by suPARnostic® ELISA kits (ViroGates) and each plate included 5% duplicates. Duplicate measurements were averaged to obtain the final concentration of biomarkers. Procedures were performed according to manufacturers’ instructions and blinded to clinical data. Biomarker concentrations outside the dynamic range were assigned the highest limit of the standard curve or a value of one-third of the lowest limit (Supplementary Table 1).

### Clinical severity scores calculation

Established clinical severity scores were selected to study relative to and in combination with biomarkers. Clinical signs collected at clinical presentation were used retrospectively to calculate these scores. The Emergency Department Paediatric Early Warning Score (ED-PEWS), the Lambaréné Organ Dysfunction Score (LODS), and the Liverpool quick Sequential Organ Failure Assessment (LqSOFA) score were used for children^21–23^. For adults, the Modified Early Warning Score (MEWS), the Quick Sequential (Sepsis-Related) Organ Failure Assessment (qSOFA) score, and the Universal Vital Assessment (UVA) score were calculated (Supplementary Table 2)^24–26^.

### Primary and secondary outcomes

The primary outcome of interest was 28-day all-cause mortality. Deaths occurring after day 28, even if captured during follow-up visits, were not considered primary outcome events. This restriction was applied to strictly define the primary outcome within a 28-day period. We conducted subgroup analyses on 28-day mortality by pre-specified patient characteristics (age group, sex, and HIV status) and restricted to inpatients. Secondary outcomes included 7-day mortality in the entire cohort, length of hospital stay in inpatients discharged home, and seeking further care and hospitalisation for the same illness up until the follow-up visit in outpatients.

### Statistical analysis

Demographic and clinical characteristics were presented as medians with interquartile ranges (IQRs) for non-normally distributed continuous variables, whilst counts and proportions were used to describe discrete variables. Biomarker concentrations in two groups were compared using Mann–Whitney U tests. Correlation of biomarker levels with continuous variables was assessed using Spearman’s rank correlation coefficients. The ability of biomarkers to discriminate between two outcomes was evaluated using receiver operating characteristic (ROC) curves and comparing the area under the ROC curves (AUROCs). The discriminative ability of the combination of two biomarkers or a biomarker and a clinical severity score was determined by fitting multivariable logistic regression models and using predicted probabilities to compute the AUROCs. Logistic models were also used to estimate odds ratios (ORs). Biomarker data were log-transformed for inclusion in all regression models. AUROCs were compared pairwise using methods recommended by DeLong et al.^27^. To stratify our study population into risk categories, we applied sTREM-1 cut-offs previously derived with data from hospitalised febrile children in Uganda for mortality prediction: low-risk ( < 239 pg/mL), intermediate-risk (239–628 pg/mL), and high-risk ( ≥ 629 pg/mL)^10^. Based on these categories, Kaplan-Meier survival curves were plotted, mortality hazard ratios were calculated using Cox proportional hazards models, and performance metrics for predicting 28-day mortality were estimated. Interactions in Cox models were assessed using likelihood ratio tests. Aside from analyses by these three risk categories, sTREM-1 was treated as a continuous variable. For the outcome length of hospital stay, we restricted the analysis to individuals discharged home and excluded in-hospital deaths, absconders, and transfers to a higher-level hospital. We performed complete case analyses, with missing values excluded. All tests were two-tailed and *p* values < 0.05 were considered statistically significant. Stata version 16.1 (StataCorp, College Station, TX, USA) was used for statistical analysis. Graphs were created using Stata version 16.1 and R version 4.2.2 (R Core Team, Vienna, Austria).

### Ethical approval

This study was approved by the Centro de Investigação em Saúde de Manhiça (CISM) Institutional Ethics Committee (Ref. CIBS-CISM/010/2018), the Mozambican National Bioethics Committee (Ref. 447/CNBS/18), the London School of Hygiene & Tropical Medicine (LSHTM) Research Ethics Committee (Ref. 14538), and the University Health Network (UHN) Institutional Review Board (Ref. UHN REB#18-6186). All research was conducted according to the principles expressed in the Declaration of Helsinki. After a detailed explanation of the study, written informed consent was obtained from all participants or from parents/guardians of paediatric participants. Children assented if aged >12 years.

### Reporting summary

Further information on research design is available in the Nature Portfolio Reporting Summary linked to this article.

## Results

### Participant characteristics

A total of 2182 participants were enrolled in the FIEBRE study in Mozambique. Of these, 1955 (89.6%) had a plasma sample taken with enough volume available for biomarker measurement and were included in this study (Supplementary Fig. 1). In 16/1955 (0.8%) participants, suPAR could not be assessed due to insufficient plasma volume for this biomarker. Among the 1955 study participants presenting to hospital, there were 531 children and 509 adults managed as outpatients, and 509 children and 406 adults managed as inpatients (Table 1).

**Table 1:** Characteristics of enrolled paediatric and adult patients with febrile illness in southern Mozambique by age group and hospitalisation decision

	Entire cohort	Child outpatients	Child inpatients	Adult outpatients	Adult inpatients
	(N = 1955)	(N = 531)	(N = 509)	(N = 509)	(N = 406)
**Demographics**					
Female sex, n (%)	1083 (55.4%)	260 (50.0%)	220 (43.2%)	384 (75.4%)	219 (53.9%)
Age in years, median (IQR)	11.2 (3.3, 33.0)	3.6 (1.8, 6.4)	3.4 (1.5, 7.0)	30.0 (23.0, 41.0)	38.0 (29.0, 53.0)
**Site of enrolment, n (%)**					
Manhiça District Hospital	1706 (87.3%)	531 (100%)	412 (80.9%)	509 (100%)	254 (62.6%)
General José Macamo Hospital	249 (12.7%)	0 (0%)	97 (19.1%)	0 (0%)	152 (37.4%)
**Days with fever before enrolment, median (IQR)**	2 (1, 3)	1 (1, 2)	2 (1, 3)	2 (2, 3)	3 (2, 7)
**Diagnostic tests at enrolment, n (%)**					
HIV infection ^a^	507/1924 (26.4%)	14/528 (2.7%)	51/500 (10.2%)	214/496 (43.1%)	228/400 (57.0%)
Malaria RDT positivity ^b^	309/1939 (15.9%)	17/531 (3.2%)	221/501 (44.1%)	29/506 (5.7%)	42/401 (10.5%)
**Clinical severity scores, median (IQR)** ^**c**^					
ED-PEWS	8 (7, 11)	8 (7, 10)	9 (7, 16)	–	–
LODS	0 (0, 0)	0 (0, 0)	0 (0, 1)	–	–
LqSOFA	0 (0, 0)	0 (0, 0)	0 (0, 0)	–	–
MEWS	3 (1, 5)	–	–	2 (1, 3)	4 (2, 5)
qSOFA	0 (0, 1)	–	–	0 (0, 0)	1 (0, 1)
UVA	2 (0, 2)	–	–	1 (0, 2)	2 (1, 3)

*ED-PEWS* Emergency Department Paediatric Early Warning Score, *HIV* human immunodeficiency virus, *IQR* interquartile range, *LODS* Lambaréné Organ Dysfunction Score, *LqSOFA* Liverpool quick Sequential Organ Failure Assessment, *MEWS* Modified Early Warning Score, *qSOFA* Quick Sequential (Sepsis-Related) Organ Failure Assessment, *RDT* rapid diagnostic test, *UVA* Universal Vital Assessment.

^a^HIV status was considered positive if self-reported by the participant/caregiver or confirmed through HIV point-of-care testing (using antibody-detecting rapid tests) performed for all participants with unknown or negative self-reported HIV status.

^b^Based on antigen-detecting lateral flow malaria RDT, which combines detection of histidine-rich protein 2 and *Plasmodium* lactate dehydrogenase.

^c^ED-PEWS, LODS, and LqSOFA scores were calculated among children only. MEWS, qSOFA, and UVA scores were calculated among adults only.

Missing data: Days with fever before enrolment, n = 88; HIV infection, n = 31; malaria RDT positivity, n = 16; ED-PEWS, n = 65; LODS, n = 56; LqSOFA, n = 44; MEWS, n = 4; qSOFA, n = 4; UVA, n = 29.

At day 28, vital status was known for 1593/1955 (81.5%) participants (Supplementary Fig. 1, Table 2). A total of 93 deaths occurred: 72 among adult inpatients, 19 among child inpatients, and two among adult outpatients. Most inpatients who died by day 28 did so in the hospital during their initial admission (39/91, 42.9%) or after they were transferred to a higher-level hospital (31/91, 34.1%), but some died after having been discharged home (19/91, 20.9%) or having absconded (1/91, 1.1%). For one deceased inpatient, this information was unavailable (1/91, 1.1%). Median time to death was 6 days (IQR: 1–12), with 28 (30.1%) deaths happening within the first 48 hours and 54 (58.1%) deaths within the first 7 days. Key clinical characteristics of paediatric and adult participants are presented separately by age group and by vital status at day 28 in Supplementary Tables 3, 4.

**Table 2:** Characteristics of enrolled paediatric and adult patients with febrile illness in southern Mozambique by vital status at day 28

	Alive at day 28	Dead by day 28
	(N = 1500)	(N = 93)
**Sex, n (%)**		
Female	843 (94.6%)	48 (5.4%)
Male	657 (93.6%)	45 (6.4%)
**Age in years**		
Median (IQR)	10.4 (3.2, 31.0)	37.0 (24.0, 56.0)
**Patient group, n (%)**		
Child outpatients	426 (100%)	0 (0%)
Child inpatients	395 (95.4%)	19 (4.6%)
Adult outpatients	426 (95.5%)	2 (0.5%)
Adult inpatients	253 (77.9%)	72 (22.2%)
**Site of enrolment, n (%)**		
Manhiça District Hospital	1366 (95.9%)	58 (4.1%)
General José Macamo Hospital	134 (79.3%)	35 (20.7%)
**Days with fever before enrolment**		
Median (IQR)	2 (1, 3)	3 (2, 7)
**HIV status, n (%)** ^**a**^		
Positive	369 (87.0%)	55 (13.0%)
Negative	1107 (96.9%)	36 (3.2%)
**Malaria status by RDT, n (%)** ^**b**^		
Positive	246 (99.6%)	1 (0.4%)
Negative	1245 (93.3%)	89 (6.7%)
**ED-PEWS** ^**c**^		
Median (IQR)	8 (7, 11)	24 (16, 35)
**LODS** ^**c**^		
Median (IQR)	0 (0, 0)	1 (0, 2)
**LqSOFA** ^**c**^		
Median (IQR)	0 (0, 0)	1 (0, 1)
**MEWS** ^**c**^		
Median (IQR)	3 (1, 4)	5 (3, 6)
**qSOFA** ^**c**^		
Median (IQR)	0 (0, 1)	1 (1, 2)
**UVA** ^**c**^		
Median (IQR)	2 (0, 2)	3 (2, 5)

*ED-PEWS *Emergency Department Paediatric Early Warning Score, *HIV *human immunodeficiency virus, *IQR *interquartile range, *LODS *Lambaréné Organ Dysfunction Score, *LqSOFA *Liverpool quick Sequential Organ Failure Assessment, *MEWS *Modified Early Warning Score, *qSOFA *Quick Sequential (Sepsis-Related) Organ Failure Assessment, *RDT *rapid diagnostic test, *UVA *Universal Vital Assessment.

^a^HIV status was considered positive if self-reported by the participant/caregiver or confirmed through HIV point-of-care testing (using antibody-detecting rapid tests) performed for all participants with unknown or negative self-reported HIV status.

^b^Based on antigen-detecting lateral flow malaria RDT, which combines detection of histidine-rich protein 2 and *Plasmodium* lactate dehydrogenase.

^c^ED-PEWS, LODS, and LqSOFA scores were calculated among children only. MEWS, qSOFA, and UVA scores were calculated among adults only.

Missing data: Days with fever before enrolment, n = 76; HIV status, n = 26; malaria RDT status by RDT, n = 12; ED-PEWS, n = 47; LODS, n = 42; LqSOFA, n = 34; MEWS, n = 3; qSOFA, n = 3; UVA, n = 26.

### Study population biomarker levels

**Fig. 1:**
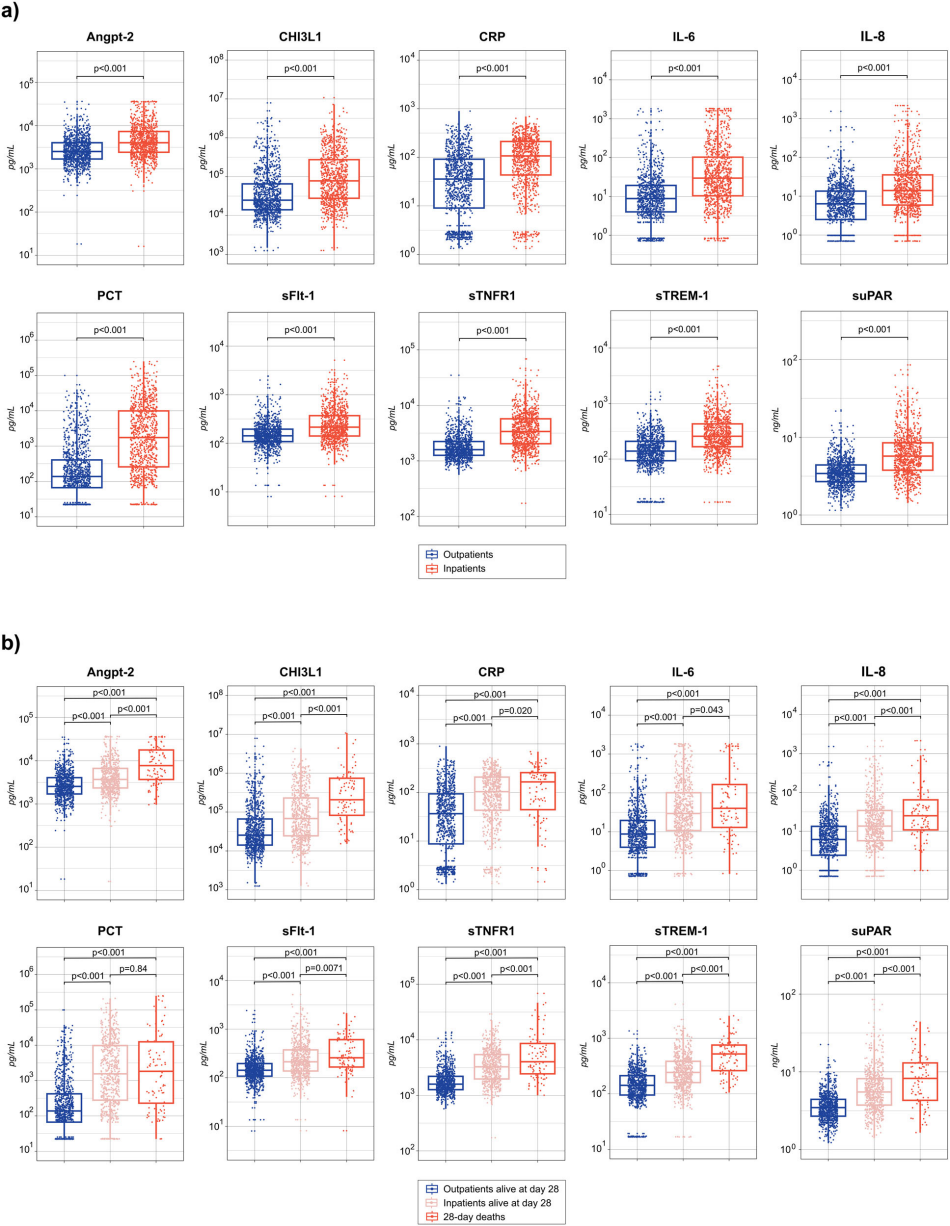
Plasma concentrations of host biomarkers at clinical presentation among enrolled paediatric and adult patients with febrile illness in southern Mozambique. Panel **a** shows biomarker concentrations in all outpatients (N = 1040) and inpatients (N = 915). Panel **b** shows biomarker concentrations in outpatients alive at day 28 (N = 852), inpatients alive at day 28 (N = 648), and deaths within 28 days (N = 93). Boxplots display the median and interquartile range, with whiskers extending to 1.5 times the interquartile range. Concentrations are in pg/mL, except for CRP (μg/mL) and suPAR (ng/mL). suPAR values are missing for 16 participants in Panel **a** and for 12 participants in Panel **b**. *p* values were calculated using Mann–Whitney U tests. Angpt-2 angiopoietin-2, CHI3L1 chitinase-3-like protein 1, CRP C-reactive protein, IL-6 interleukin-6, IL-8 interleukin-8, PCT procalcitonin, sFlt-1 soluble fms-like tyrosine kinase-1, sTNFR1 soluble tumour necrosis factor receptor 1, sTREM-1 soluble triggering receptor expressed on myeloid cells 1, suPAR soluble urokinase-type plasminogen activator receptor.

All biomarker levels at clinical presentation differed significantly between inpatients and outpatients, with higher levels observed in inpatients (all p < 0.001) (Fig. 1a). Among participants with a known vital status at day 28, all biomarkers were significantly elevated at presentation in participants who subsequently died compared to those who remained alive at day 28 (all p < 0.001). Participants who died also had significantly higher biomarker levels than both surviving outpatients and surviving inpatients when these groups were analysed separately, except for PCT levels when compared to surviving inpatients (Fig. 1b). Levels of biomarkers by hospitalisation and vital status at day 28 are displayed separately for children and adult participants in Supplementary Fig. 2.

### Comparative performance of biomarkers to predict 28-day mortality

**Fig. 2:**
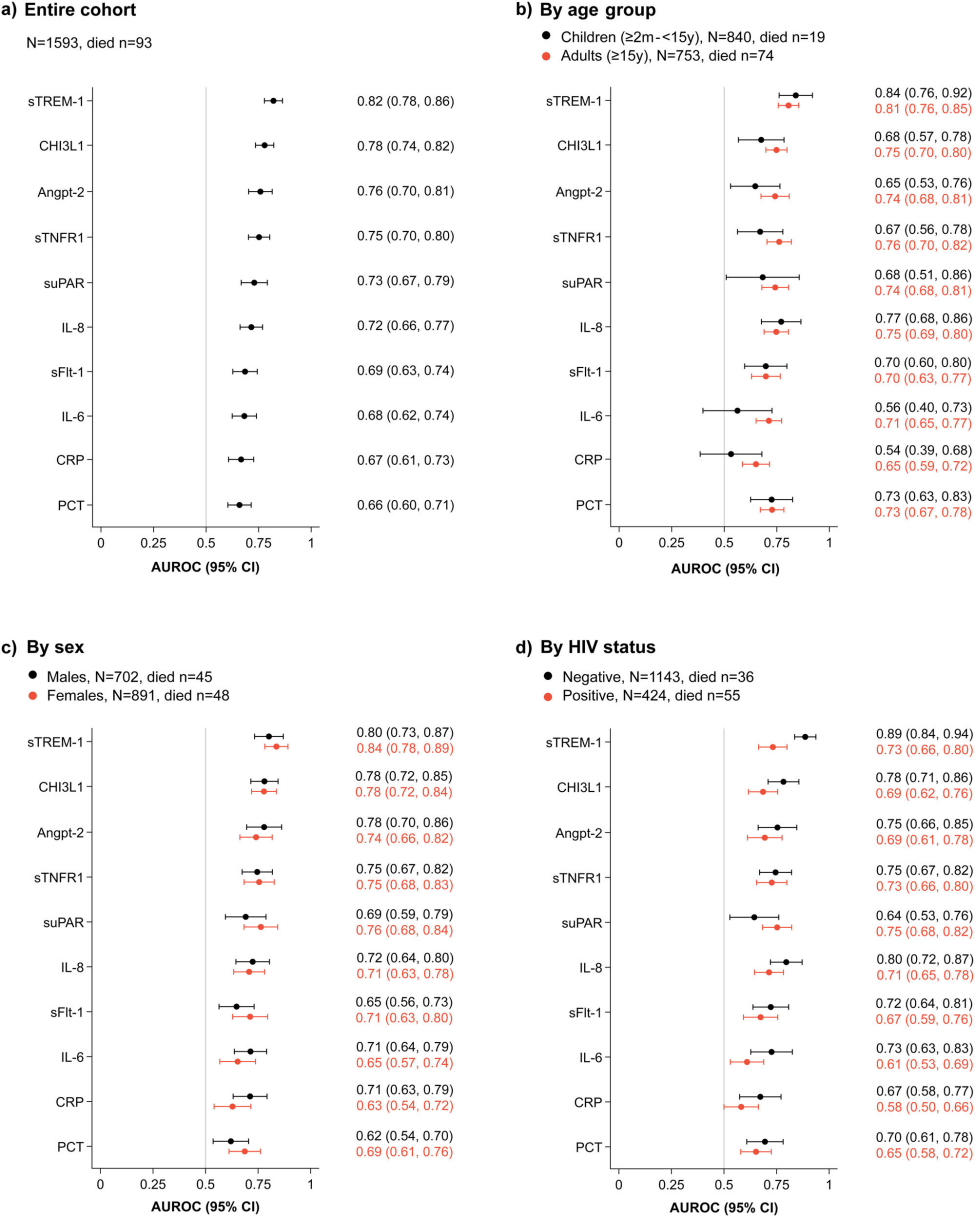
AUROC of each host biomarker for 28-day mortality among enrolled paediatric and adult patients with febrile illness in southern Mozambique. Plots display the AUROC (dot) with 95% CI (horizontal line) of each biomarker for 28-day mortality in the entire cohort (Panel **a**) and in each patient subgroup according to age group (Panel **b**), sex (Panel **c**), and HIV status (Panel **d**). Corresponding numerical values of the AUROCs, with 95% CIs in parentheses, are shown to the right of each plot. For suPAR, the total sample size is N = 1581 (91 deaths); restricting analyses to this population does not affect the overall ranking of AUROCs or the AUROC values of sTREM-1. Angpt-2 angiopoietin-2, AUROC area under the receiver operating characteristic curve, CHI3L1 chitinase-3-like protein 1, CI confidence interval, CRP C-reactive protein, HIV human immunodeficiency virus, IL-6 interleukin-6, IL-8 interleukin-8, PCT procalcitonin, sFlt-1 soluble fms-like tyrosine kinase-1, sTNFR1 soluble tumour necrosis factor receptor 1, sTREM-1 soluble triggering receptor expressed on myeloid cells 1, suPAR soluble urokinase-type plasminogen activator receptor.

Among assessed biomarkers, sTREM-1 levels at clinical presentation best discriminated participants who subsequently died within 28 days, with an AUROC of 0.82 (95% confidence interval [CI]: 0.78–0.86) (Fig. 2a). sTREM-1 was superior in terms of AUROCs when compared individually to PCT, CRP, IL-6, sFlt-1, and IL-8 (all p < 0.001), as well as to suPAR and sTNFR1 (p = 0.0070 and p = 0.0030, respectively). With univariable logistic regression, the odds of 28-day mortality increased by 3.19 times (95% CI: 2.57-3.96) for every two-fold increase in sTREM-1 concentration (Supplementary Table 5). The best two-biomarker combination by AUROC for 28-day mortality was sTREM-1 and Angpt-2, with an AUROC of 0.85 (95% CI: 0.81–0.88) (p = 0.054 compared to that of sTREM-1 alone). Among inpatients only, sTREM-1 had the best discriminative ability for 28-day mortality with an AUROC of 0.74 (95% CI: 0.68–0.79) (Supplementary Fig. 3). When limiting the outcome to 7-day mortality, sTREM-1 remained the best biomarker with an AUROC of 0.82 (95% CI: 0.77–0.88) (Supplementary Fig. 4).

We evaluated the discriminative ability of all biomarkers for 28-day mortality across different patient subgroups (Fig. 2b–d). sTREM-1 had the highest AUROC irrespective of age group and sex. By HIV status, sTREM-1 had the highest AUROC for HIV-negative individuals and the second highest for HIV-positive individuals after suPAR. Comparisons of AUROCs for each biomarker between subgroups showed no significant differences, except for sTREM-1 by HIV status. The AUROC of sTREM-1 was higher for HIV-negative participants (0.89, 95% CI: 0.84–0.94) compared to HIV-positive participants (0.73, 95% CI: 0.66-0.80) (p < 0.001). Among HIV-positive participants, the AUROC of sTREM-1 in those who reported taking antiretroviral therapy (ART) was 0.77 (95% CI: 0.69–0.84) and in those who did not or were newly diagnosed was 0.64 (95% CI: 0.49–0.78). sTREM-1 levels according to vital status at day 28 and HIV status are plotted in Supplementary Fig. 5.

### Mortality risk stratification using sTREM-1 cut-offs

**Fig. 3:**
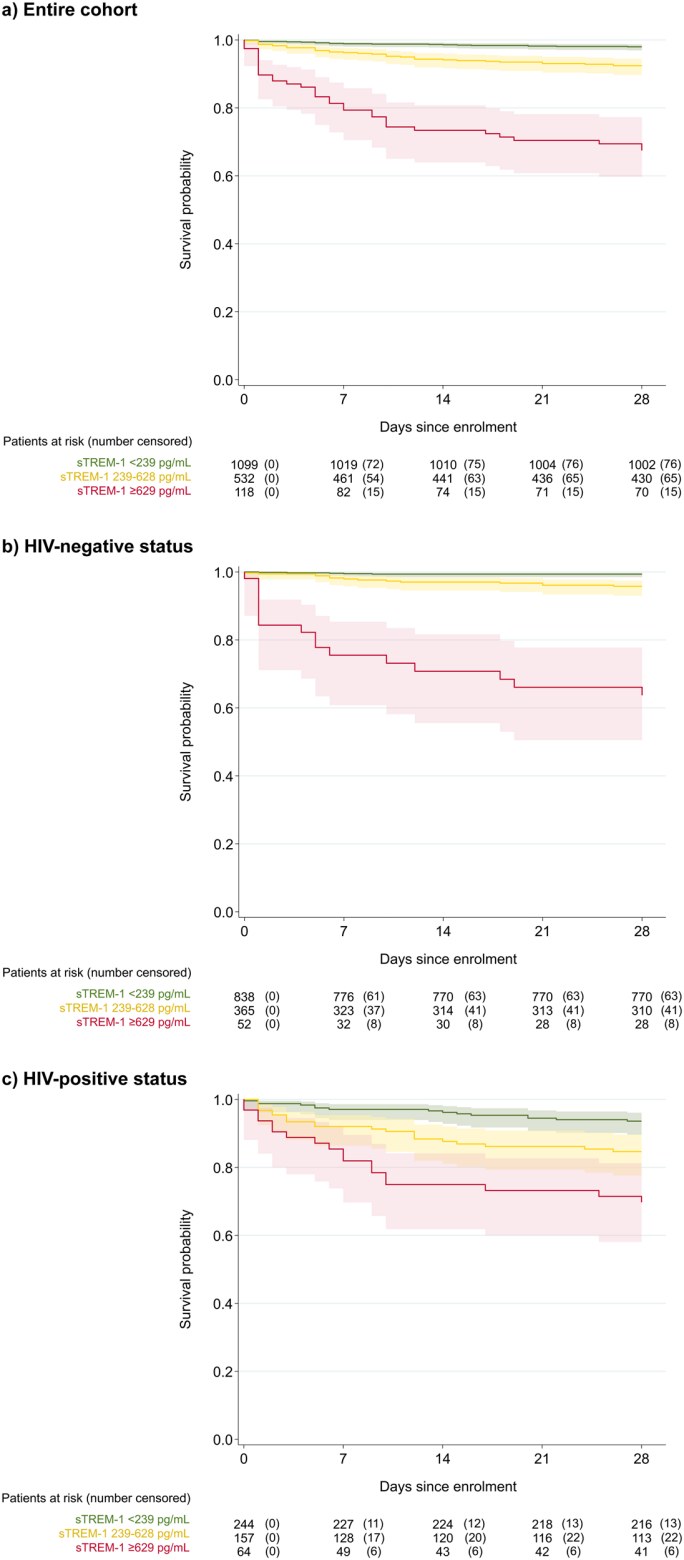
Kaplan-Meier survival curves by sTREM-1 categories among enrolled paediatric and adult patients with febrile illness in southern Mozambique. Kaplan-Meier curves with 95% CIs illustrate the survival probability over time by sTREM-1 categories for the entire cohort (Panel **a**), HIV-negative individuals (Panel **b**), and HIV-positive individuals (Panel **c**). We applied sTREM-1 cut-offs previously derived with data from hospitalized febrile children in Uganda for mortality prediction^10^, stratifying participants into three risk categories based on sTREM-1 levels: low-risk (sTREM-1 < 239 pg/mL), intermediate-risk (sTREM-1 239–628 pg/mL), and high-risk (sTREM-1 ≥ 629 pg/mL). Inpatients with unknown vital status at day 28 were included in the analyses and censored when discharged alive from hospital. CI confidence interval, HIV human immunodeficiency virus, sTREM-1 soluble triggering receptor expressed on myeloid cells 1.

Using previously defined sTREM-1 cut-offs^10^, 64.6% of participants were classified as low-risk (80.9% of outpatients vs. 46.0% of inpatients), 29.0% as intermediate-risk (18.0% of outpatients vs. 41.4% of inpatients), and 6.5% as high-risk (1.2% of outpatients vs. 12.6% of inpatients). This corresponded to a 28-day case fatality ratio of 34.0% (35/103) for the high-risk group, 7.9% (37/467) for the intermediate-risk group, and 2.1% (21/1,023) for the low-risk group. Supplementary Table 6 presents the sensitivity, specificity, and other performance metrics for predicting 28-day mortality using these sTREM-1 cut-offs. Mortality hazard ratios were 19.5 (95% CI: 11.3–33.5) and 3.9 (95% CI: 2.3–6.6) in the high-risk and intermediate-risk categories compared to the low-risk category, respectively (Fig. 3a). HIV status was an effect modifier of the association between sTREM-1-based risk category and mortality (p < 0.001) (Fig. 3b, c). Among the 21 deaths in the low-risk group, 15 (71.4%) were HIV-positive and 1 (4.8%) had an unknown HIV status. However, there was no effect modification by age group or sex (Supplementary Fig. 6).

### Performance of sTREM-1 relative to lactate to predict 28-day mortality

A subsample of participants had documented lactate levels at clinical presentation and a known vital status at day 28 (N = 1190). Lactate was associated with 28-day mortality (p < 0.001) and exhibited an AUROC of 0.72 (95% CI: 0.66-0.79) for 28-day mortality. This was outperformed by the sTREM-1 AUROC of 0.83 (95% CI: 0.78–0.88) (p = 0.0033). AUROCs of sTREM-1 and lactate by patient subgroups can be found in Supplementary Fig. 7.

### Performance of sTREM-1 relative to and in combination with clinical severity scores to predict 28-day mortality

**Fig. 4:**
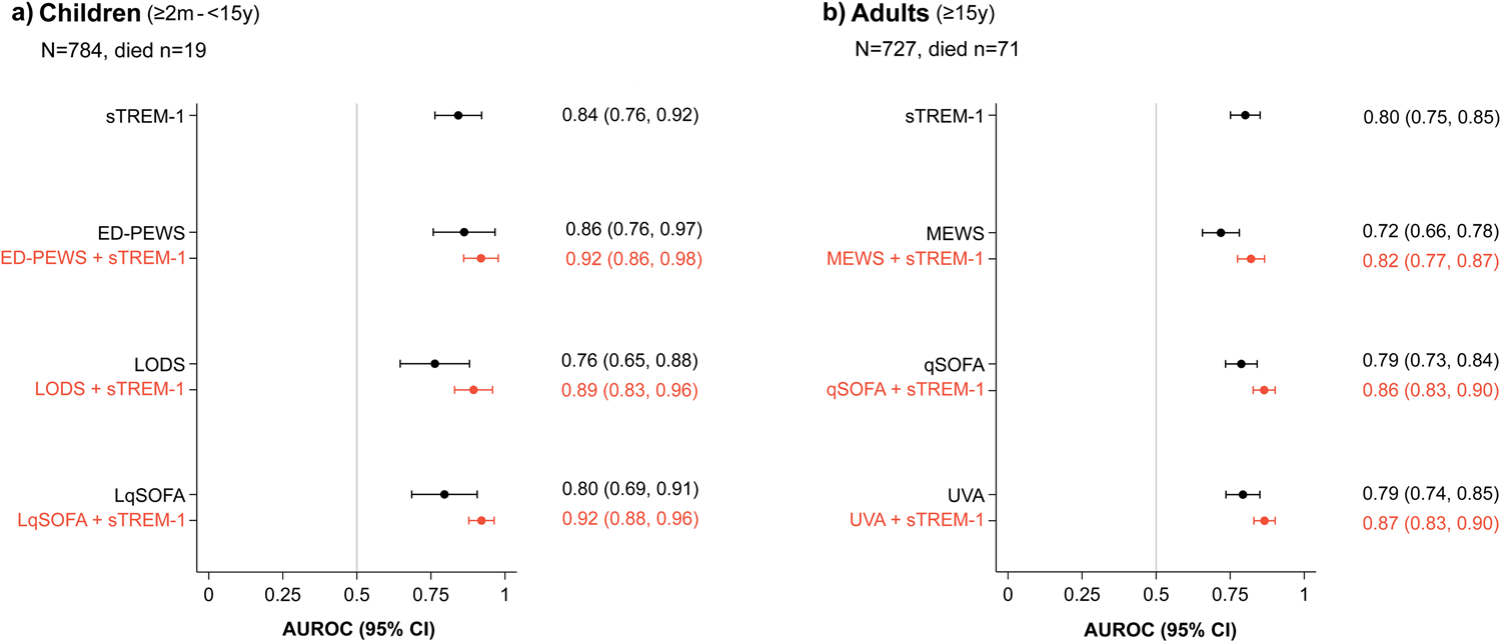
AUROC of each clinical severity score alone and combined with sTREM-1 for 28-day mortality among enrolled paediatric and adult patients with febrile illness in southern Mozambique. Plots display the AUROC (dot) with 95% CI (horizontal line) of sTREM-1, each clinical severity score, and the combination of sTREM-1 and each clinical severity score for 28-day mortality in children (Panel **a**) and in adults (Panel **b**). Corresponding numerical values of the AUROCs, with 95% CIs in parentheses, are shown to the right of each plot. We included all participants with a known vital status at day 28 and clinical data for the calculation of all clinical severity scores. AUROC area under the receiver operating characteristic curve, CI confidence interval, ED-PEWS Emergency Department Paediatric Early Warning Score, LODS Lambaréné Organ Dysfunction Score, LqSOFA Liverpool quick Sequential Organ Failure Assessment, MEWS Modified Early Warning Score, qSOFA Quick Sequential (Sepsis-Related) Organ Failure Assessment, sTREM-1 soluble triggering receptor expressed on myeloid cells 1, UVA Universal Vital Assessment.

Different clinical severity scores were calculated for study participants (Supplementary Fig. 8). A one-unit increase in all scores was associated with increased odds of 28-day mortality (all p < 0.001). However, sTREM-1 remained significantly associated with 28-day mortality when adjusted for each individual score (Supplementary Table 7). ED-PEWS and UVA were the scores with the best AUROCs for 28-day mortality in children and adults, respectively (Fig. 4a-b). sTREM-1 alone demonstrated a comparable AUROC to clinical severity scores, with a superior AUROC to MEWS in adults (p = 0.016). The combination of sTREM-1 with these scores significantly improved the AUROCs of all models in adults (p = 0.0013 for UVA, p < 0.001 for MEWS and for qSOFA) and of two models in children (p = 0.0084 for LODS, p = 0.0096 for LqSOFA, p = 0.19 for ED-PEWS) (Fig. 4a-b).

### Biomarkers and adverse outcomes other than mortality

The median duration of hospitalisation for inpatients discharged home was three days (IQR: 1–5). sTREM-1, as well as Angpt-2 and CHI3L1, were positively correlated with length of hospital stay (all p < 0.001). Of these, sTREM-1 had the highest positive Spearman’s rank correlation coefficient (Table 3).

**Table 3:** Association of host biomarker levels in plasma at clinical presentation with different adverse outcomes among enrolled paediatric and adult patients with febrile illness in southern Mozambique

	Length of hospital stay in inpatients discharged home (N = 675)		Seeking further care for the same illness by the follow-up visit in outpatients (N = 838) ^a^			Subsequent hospitalisation for the same illness by the follow-up visit in outpatients (N = 838) ^a^		
Biomarker	Spearman’s rank correlation coefficient (ρ)	p value	Did not seek care (N = 780), median (IQR)	Sought care (N = 58), median (IQR)	p value	No hospitalisation (N = 828), median (IQR)	Hospitalisation (N = 10), median (IQR)	p value
Angpt-2	0.18	<0.001	2552.2 (1680.9, 4023.1)	2579.6 (1928.4, 4529.8)	0.24	2537.5 (1703.2, 4037.1)	4243.1 (2522.3, 6634.0)	0.14
CHI3L1	0.24	<0.001	24280.0 (13879.7, 67348.4)	28446.8 (17469.3, 60298.5)	0.17	24739.2 (14070.8, 66283.2)	50972.3 (33465.4, 172075.8)	0.11
CRP	0.01	0.77	35.6 (8.7, 91.8)	52.8 (16.6, 121.5)	0.083	36.0 (8.7, 93.1)	79.0 (55.7, 189.6)	0.012
IL-6	–0.03	0.43	8.5 (4.0, 19.5)	11.6 (5.0, 26.9)	0.12	8.6 (4.0, 19.6)	17.6 (12.3, 33.3)	0.0079
IL-8	0.05	0.17	6.2 (2.3, 13.5)	5.7 (3.3, 16.0)	0.45	6.1 (2.4, 13.5)	9.5 (6.8, 44.4)	0.080
PCT	–0.07	0.080	128.9 (45.7, 396.8)	234.3 (89.7, 1029.5)	0.0056	131.7 (66.3, 405.2)	1011.6 (487.1, 3113.5)	0.0010
sFlt-1	–0.10	0.010	139.7 (106.7, 193.4)	155.3 (116.3, 221.6)	0.055	142.1 (107.7, 195.2)	175.4 (107.2, 221.6)	0.31
sTNFR1	0.00	0.94	1574.1 (1256.6, 2166.1)	1711.7 (1445.8, 2532.2)	0.011	1584.3 (1264.9, 2180.8)	2672.3 (1752.6, 4312.3)	0.0014
sTREM-1	0.34	<0.001	138.9 (93.4, 208.2)	168.6 (123.5, 257.8)	0.0022	140.4 (94.2, 210.2)	214.4 (155.8, 371.3)	0.012
suPAR	0.01	0.88	3.4 (2.7, 4.4)	4.1 (3.2, 5.3)	0.0035	3.5 (2.7, 4.4)	5.4 (4.2, 8.9)	0.0015

*Angpt-2 *angiopoietin-2, *CHI3L1 *chitinase-3-like protein 1, *CRP *C-reactive protein, *IL-6 *interleukin-6, *IL-8 *interleukin-8, *IQR *interquartile range, *PCT *procalcitonin, *sFlt-1 *soluble fms-like tyrosine kinase-1, *sTNFR1 *soluble tumour necrosis factor receptor 1, *sTREM-1 *soluble triggering receptor expressed on myeloid cells 1, *suPAR *soluble urokinase-type plasminogen activator receptor.

^a^Concentrations are in pg/mL, except for CRP (μg/mL) and suPAR (ng/mL). p values for comparisons of medians were calculated using Mann–Whitney U tests.

Missing data: All included patients had complete biomarker data.

Among the 838 outpatients with follow-up data at day ≥28, 780 (93.1%) did not seek additional care for the same illness, 48 (5.7%) sought further care but were not hospitalised, and 10 (1.2%) sought further care and required hospitalisation. sTREM-1, suPAR, PCT, and sTNFR1 levels at presentation were significantly higher in outpatients who subsequently sought further care (Table 3). sTREM-1 levels, along with most assessed biomarkers, were significantly higher in outpatients who were later hospitalised (Table 3). sTREM-1 had an AUROC of 0.62 (95% CI: 0.55–0.69) for seeking further care and of 0.73 (95% CI: 0.60–0.87) for subsequent hospitalisation in outpatients (Supplementary Table 8).

## Discussion

In this cohort of Mozambican children and adults with febrile illness presenting to hospital, sTREM-1 was the best biomarker for predicting 28-day mortality. sTREM-1 was superior to other biomarkers of immune and endothelial activation and provided additional discriminative ability when combined with clinical severity scores. Notably, sTREM-1 consistently demonstrated the best performance across different patient subgroups, though its performance was diminished in HIV-positive individuals. sTREM-1 remained the best biomarker if 7-day mortality was considered instead. sTREM-1 was positively correlated with length of hospital stay and was also the best biomarker to predict mortality among inpatients. Outpatients had lower plasma concentrations of all studied biomarkers compared to inpatients, yet their sTREM-1 levels were associated with seeking further care and subsequent hospitalisation.

These results provide additional evidence supporting the use of sTREM-1 as a predictor of mortality in children and adults with all-cause febrile illness. In hospitalised febrile children in Uganda, sTREM-1 had the best prognostic performance for 7-day mortality among a similar biomarker panel, with an AUROC of around 0.90^10^. Likewise, in Tanzanian febrile adults presenting to outpatient clinics, sTREM-1 was the best biomarker to predict 28-day mortality with an AUROC of 0.87 (95% CI: 0.81–0.92)^11^. In diverse populations with specific syndromes or detected infectious agents (including malaria, pneumonia, COVID-19, or sepsis), high sTREM-1 levels have been consistently associated with subsequent mortality^12–15,28–31^. When we applied previously defined sTREM-1 cut-offs for risk stratification, we observed a stepwise increase in mortality across categories, similar to that reported in other cohorts^10,13,15^. Considering the challenges in determining the underlying cause of febrile illnesses, these data collectively suggest the broad applicability of sTREM-1 to risk-stratify febrile patients in a pathogen-agnostic manner.

sTREM-1 significantly outperformed CRP and PCT in predicting 28-day mortality, consistent with earlier findings from similar populations^10,11^. sTREM-1 also outperformed lactate. Although prior research on pneumonia, malaria, and sepsis has documented an association between lactate levels and fatal outcome, the prognostic performance of lactate was usually inferior to sTREM-1^12,14,32,33^. In addition, we applied various relevant clinical severity scores to our study population, similarly to previous publications using data from the FIEBRE study^34^, and compared them to sTREM-1. Comprehensive clinical data collection occurred under our study conditions; however, it is worth noting that some variables may be measured with less accuracy or be unavailable in routine clinical practice. sTREM-1 alone had similar discriminative ability for 28-day mortality when compared to clinical severity scores, and the addition of sTREM-1 added discriminative ability to the scores. This result, coupled with similar previous findings, suggests that sTREM-1 could be integrated with relevant and context-specific clinical severity scores or decision algorithms to better risk-stratify patients with suspected acute infections^11,13,15,28,35^. CHI3L1 and Angpt-2 are other candidate biomarkers that had comparable performance to sTREM-1 overall, but not across all patient subgroups. Combining biomarkers could enhance mortality prediction, although this may limit feasibility and practicality for clinical use. sTREM-1 and Angpt-2 was the best two-biomarker combination to predict 28-day mortality based on AUROC, but adding Angpt-2 only slightly increased the AUROC over sTREM-1 alone.

The prognostic performance of sTREM-1 for 28-day mortality differed by HIV status. While sTREM-1 remained a good predictor in HIV-positive participants, its performance was significantly inferior in this group compared to HIV-negative participants. This was more notable in individuals not receiving ART or unaware of their HIV-positive status. Active HIV infection causes a combination of immunosuppression and chronic inflammation that can result in immune system exhaustion and altered host responses to pathogens^36^. Most of the deaths in the sTREM-1-based low-risk group were HIV-positive. Moreover, previous research has linked HIV infection with upregulation of TREM-1 in immune cells and increased plasma levels of sTREM-1, suggesting a possible contribution of TREM-1 to HIV-induced chronic inflammation, and hence potentially diminishing the prognostic performance of sTREM-1 in acute infections^37,38^. One previous study in children with signs of severe infection explored sTREM-1 levels in relation to HIV status and mortality, but in this cohort sTREM-1 was not associated with mortality regardless of HIV status^39^. Additional research is needed to elucidate the mechanistic impact of HIV infection on the TREM-1 pathway during febrile illnesses. Except for sTREM-1 and HIV status, selected biomarkers showed similar prognostic performance for 28-day mortality across patient subgroups. Nevertheless, almost all biomarkers tended to have lower AUROCs in HIV-positive participants. Existing limited evidence in febrile children found no sex-related differences in biomarker levels in relation to mortality^40^. However, in paediatric severe malaria, sTREM-1 and sFlt-1 predicted mortality better in males^15^. PCT and IL-6 were inferior at predicting poor outcome in infants compared to older children with pneumonia in Bhutan^35^. Future studies are needed to examine potential differences in biomarker prognostic performance across patient characteristics.

Death is often the primary outcome in prognostic studies with inpatients. Yet associations with less severe outcomes are also important for patient management, particularly in lower mortality risk clinical settings. In this cohort, mortality among outpatients was very low, and other adverse outcomes were assessed within this subgroup. Along with other biomarkers, we demonstrated an association between sTREM-1 levels and seeking additional care or subsequent hospitalisation in outpatients, suggesting that the studied biomarkers could play a future role in mitigating these adverse outcomes. Previous evidence on sTREM-1 and adverse outcomes in patients attending outpatient clinics and emergency departments is scarce. In these contexts, sTREM-1 has been associated with hospital admission and with mortality, in line with our results^11,30^. However, in Spanish outpatients aged over 50 years with mild-to-moderate COVID-19, sTREM-1 poorly predicted hospitalization by day 28. This contrasts with our study findings, likely due to differences in demographics, disease severity, and healthcare settings^41^. In addition, sTREM-1 has been reported to be less suitable for predicting supplementary oxygen requirement in respiratory diseases^42–44^. Further research is necessary to better understand whether sTREM-1 is sufficiently altered early in the disease course and confirm its prognostic utility for other adverse outcomes beyond mortality.

sTREM-1 has a pathophysiologic link with sepsis. TREM-1 is a cell-surface receptor expressed mostly by myeloid immune cells, which amplifies inflammation in infections^45^. sTREM-1 acts as a decoy receptor and dampens TREM-1 activation^45^. It is thought that sTREM-1 release depends on TREM-1 pathway activation and that it counteracts excessive inflammatory reactions, but high levels may reflect an underlying immune dysfunction^46,47^. Interestingly, efforts have been made to modulate TREM-1 amplification in severe infections^47^. Recent clinical trials in septic shock and COVID-19 have demonstrated that nangibotide, a TREM-1 specific inhibitor, was safe and holds potential to improve clinical status^48,49^. Therefore, risk stratification strategies based on sTREM-1 could be coupled with targeted therapeutic strategies acting on the same pathways.

This study had several strengths, including its prospective enrolment of participants, a large and well-characterized cohort comprising both inpatients and outpatients, and a head-to-head comparison of several candidate biomarkers described in the literature. However, it also has limitations. The stratified enrolment strategy resulted in the inclusion of a high proportion of patients requiring hospitalisation, potentially limiting the representativeness with regard to all febrile cases presenting at the recruiting hospitals. There was a considerable loss to follow-up for the 28-day visit. Although this study had a significant sample size and number of primary outcome events, the statistical power for some subgroup or secondary analyses was limited. Additionally, we lacked detailed information on barriers to seeking healthcare and when and where outpatients sought further care or were hospitalised, which could have provided further insights and allowed us to restrict events stringently to 28 days as we did with mortality. Furthermore, information on viral load or CD4 cell count for HIV-positive participants was unavailable, which would have better indicated participants with HIV-associated immunosuppression. Conversely, we relied on self-reported data regarding ART use and awareness of a previous HIV diagnosis. We also lacked data on HIV status confirmation by molecular methods for seropositive children aged <18 months and they were considered positive cases. All studied biomarkers, except lactate, were measured retrospectively on stored samples, which might have given slightly different results than if all the testing had been done at the point of care.

In conclusion, this study supports sTREM-1 as a robust predictor of mortality in both children and adults with all-cause fever and provides evidence of its association with other adverse outcomes. Moreover, it shows that sTREM-1 prognostic performance may be diminished in HIV-positive individuals. While its application in different clinical scenarios warrants further investigation through interventional studies, measuring sTREM-1 in patients with febrile illness in resource-limited settings may facilitate timely risk stratification and management decisions with a positive impact on clinical outcomes.

## Supplementary information

Supplementary information

Reporting summary

## Data Availability

The de-identified dataset, along with the corresponding data dictionary that defines each field in the set, is freely available with no restrictions via LSHTM’s Data Compass and can be accessed and downloaded at 10.17037/DATA.00004583^50^ The numerical data used to plot all figures are available at the same location.
