# Supplementary material for: Soluble triggering receptor expressed on myeloid cells 1 (sTREM-1) predicts mortality in patients with febrile illness in southern Mozambique
Source: Commun Med (Lond). 2025 Jul 25;5:310. doi: 10.1038/s43856-025-01014-2 (PMC12297511; doi:10.1038/s43856-025-01014-2)
Supplement: Supplementary file 1 — Supplementary information [file 43856_2025_1014_MOESM1_ESM.pdf]

Soluble triggering receptor expressed on myeloid cells 1 (sTREM-1) predicts mortality in patients with febrile illness in southern Mozambique

SUPPLEMENTARY INFORMATION

Núria Balanza, Bàrbara Baro, Sara Ajanovic, Zumilda Boca, Justina Bramugy, Anelsio Cossa, Elizabeth JA. Fitchett, Heidi Hopkins, Suzanne H. Keddie, Sham Lal, David C. W. Mabey, Tegwen Marlais, Hridesh Mishra, Campos Mucasse, Marta Valente, Andrea M. Weckman, Julie K. Wright, Shunmay Yeung, Kathleen Zhong, Kevin C. Kain, Quique Bassat

<u>**TABLE OF CONTENTS**</u>

I. SUPPLEMENTARY FIGURES.. 2

Supplementary Figure 1. Flowchart of study participants.. 2

Supplementary Figure 2. Plasma concentrations of host biomarkers at clinical presentation among enrolled paediatric or adult patients with febrile illness in southern Mozambique by hospitalisation decision and vital status at day 28 3

Supplementary Figure 3. AUROC of each host biomarker for 28-day mortality among enrolled paediatric and adult inpatients with febrile illness in southern Mozambique 4

Supplementary Figure 4. AUROC of each host biomarker for 7-day mortality among enrolled paediatric and adult patients with febrile illness in southern Mozambique 5

Supplementary Figure 5. sTREM-1 concentrations by vital status at day 28 and HIV status among enrolled paediatric and adult patients with febrile illness in southern Mozambique 6

Supplementary Figure 6. Kaplan-Meier survival curves by sTREM-1 categories among different patient subgroups of enrolled paediatric and adult patients with febrile illness in southern Mozambique.. 7

Supplementary Figure 7. AUROC of sTREM-1 and lactate for 28-day mortality among enrolled paediatric and adult patients with febrile illness in southern Mozambique 8

Supplementary Figure 8. The percentage distribution of each clinical severity score and associated 28-day mortality among enrolled paediatric or adult patients with febrile illness in southern Mozambique.. 9

II. SUPPLEMENTARY TABLES 10

Supplementary Table 1. Percentage and distribution of biomarker values outside the dynamic range................. 10

Supplementary Table 2. Components of the clinical severity scores and their threshold values and points allocated 11

Supplementary Table 3. Demographic and clinical characteristics of enrolled paediatric patients with febrile illness in southern Mozambique by vital status at day 28.. 12

Supplementary Table 4. Demographic and clinical characteristics of enrolled adult patients with febrile illness in southern Mozambique by vital status at day 28.. 13

Supplementary Table 5. Association of host biomarkers with 28-day mortality from univariable logistic regression among enrolled paediatric and adult patients with febrile illness in southern Mozambique 14

Supplementary Table 6. Performance metrics of sTREM-1 cut-offs for predicting 28-day mortality among enrolled paediatric and adult patients with febrile illness in southern Mozambique 15

Supplementary Table 7. Association of sTREM-1 and clinical severity scores with 28-day mortality from univariable and multivariable logistic regression among enrolled paediatric or adult patients with febrile illness in southern Mozambique.. 16

Supplementary Table 8. AUROC of each host biomarker for predicting adverse outcomes other than mortality among enrolled paediatric and adult outpatients with febrile illness in southern Mozambique 17

III. SUPPLEMENTARY REFERENCES 18

I. SUPPLEMENTARY FIGURES

Supplementary Figure 1. Flowchart of study participants

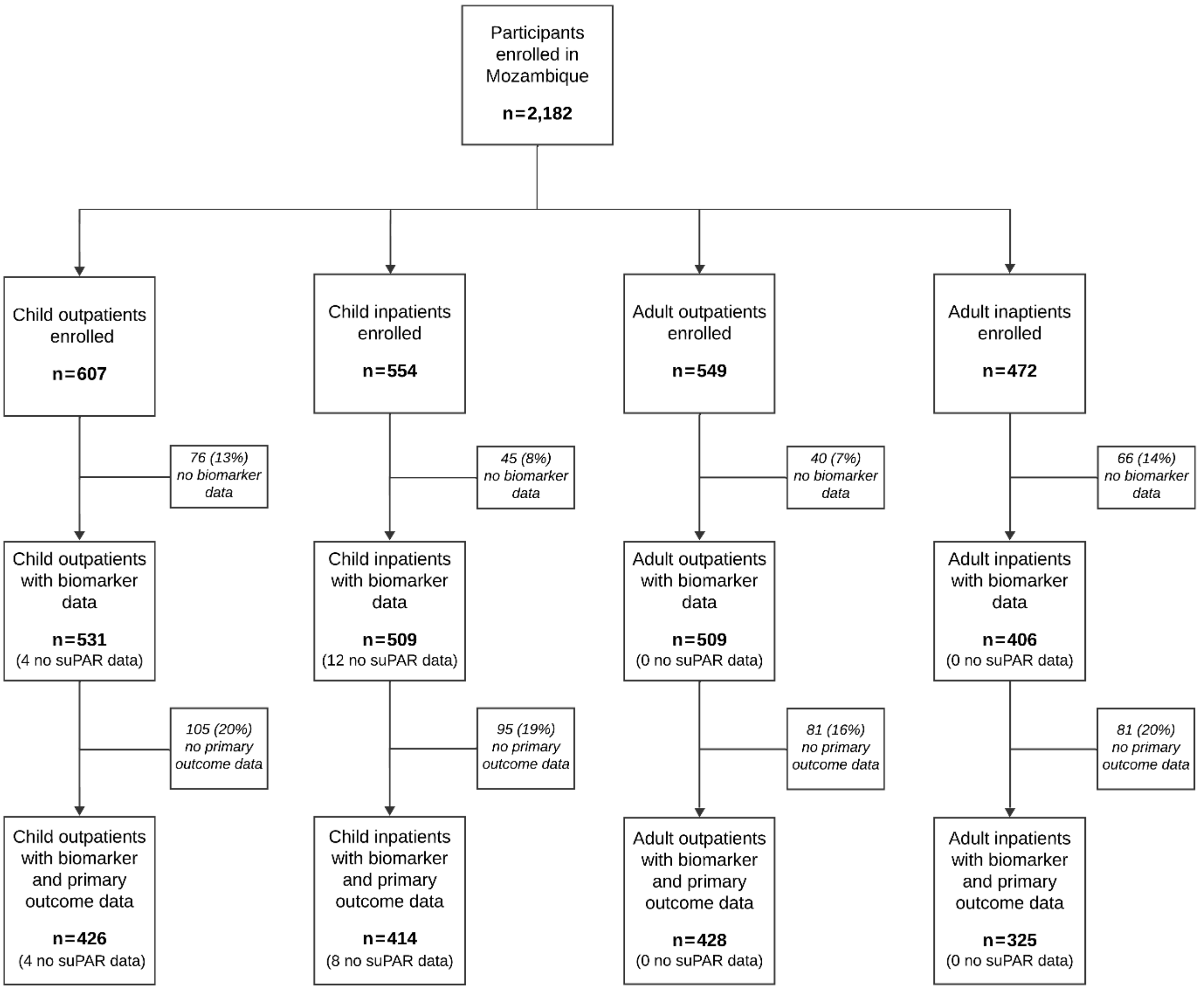

Supplementary Figure 2. Plasma concentrations of host biomarkers at clinical presentation among enrolled paediatric or adult patients with febrile illness in southern Mozambique by hospitalisation decision and vital status at day 28

A) Children (≥2m - <15y)

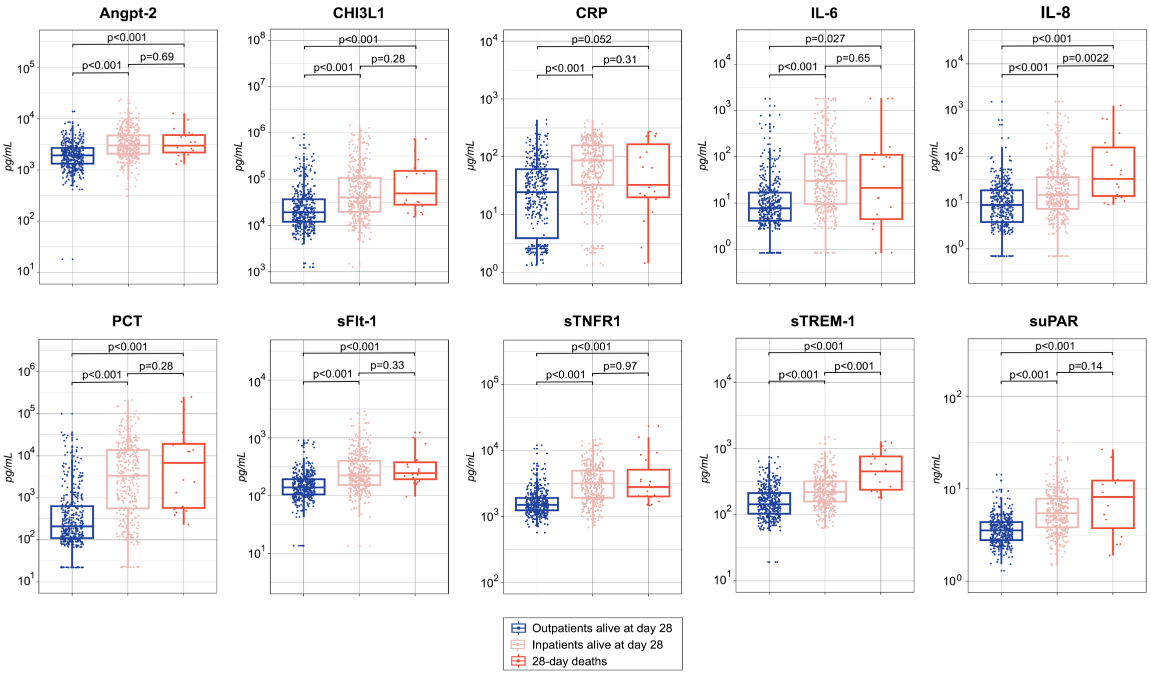

B) Adults (≥15y)

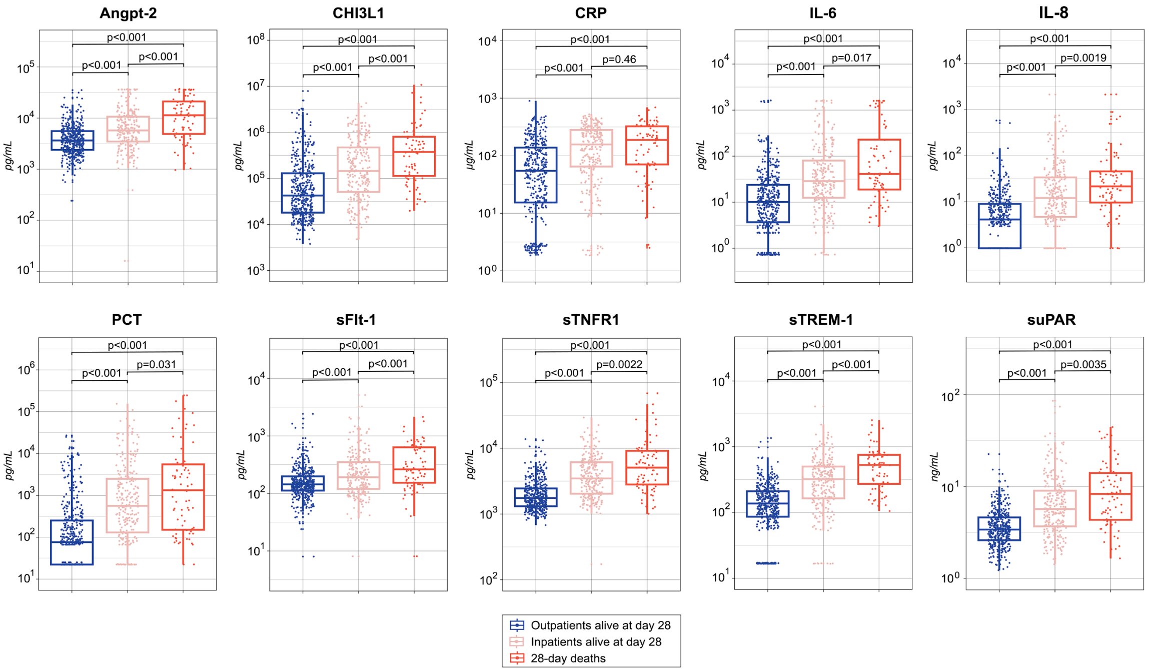

Panel A) Biomarker concentrations in outpatients alive at day 28 (N=426), inpatients alive at day 28 (N=395), and deaths within 28 days (N=19) in children. Panel B) Biomarker concentrations in outpatients alive at day 28 (N=426), inpatients alive at day 28 (N=253), and deaths within 28 days (N=74) in adults. Boxplots display the median and interquartile range, with whiskers extending to 1.5 times the interquartile range. Concentrations are in pg/mL, except for CRP (µg/mL) and suPAR (ng/mL). suPAR values are missing for 16 participants in Panel A. p-values were calculated using Mann-Whitney U tests.

Abbreviations: Angpt-2=angiopoietin-2, CHI3L1=chitinase-3-like protein 1, CRP=C-reactive protein, IL-6=interleukin-6, IL-8=interleukin-8, PCT=procalcitonin, sFlt-1=soluble fms-like tyrosine kinase-1, sTNFR1=soluble tumour necrosis factor receptor 1, sTREM-1=soluble triggering receptor expressed on myeloid cells 1, suPAR=soluble urokinase-type plasminogen activator receptor.

Supplementary Figure 3. AUROC of each host biomarker for 28-day mortality among enrolled paediatric and adult inpatients with febrile illness in southern Mozambique

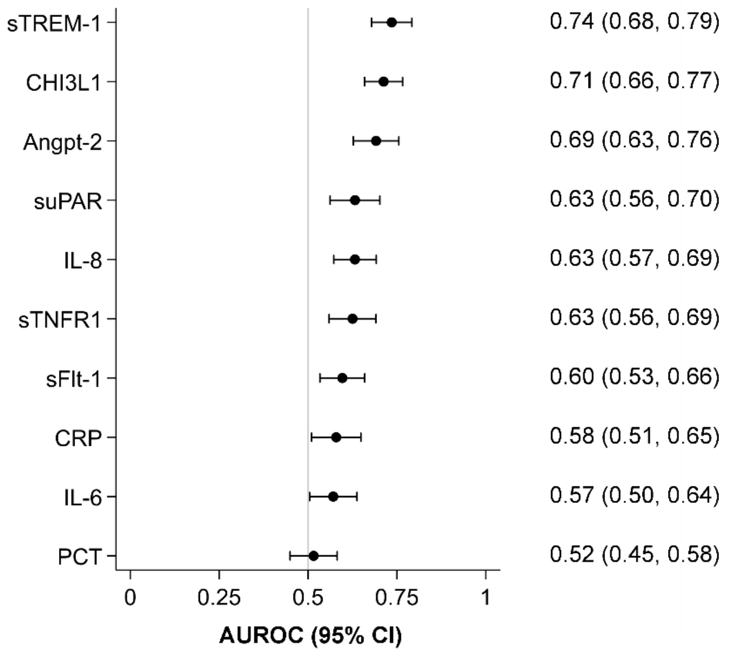

The plot displays the AUROC (dot) with 95% CI (horizontal line) of each biomarker for 28-day mortality. Corresponding numerical values of the AUROCs, with 95% CIs in parentheses, are shown to the right of the plot. The total sample size is N=739 (91 deaths). For suPAR, the total sample size is N=731 (89 deaths); restricting analyses to this population does not affect the ranking of AUROCs and the AUROC of sTREM-1 remains 0.74 (95% CI: 0.68-0.79).

Abbreviations: Angpt-2=angiopoietin-2, AUROC=area under the receiver operating characteristic curve, CHI3L1=chitinase-3-like protein 1, CI=confidence interval, CRP=C-reactive protein, IL-6=interleukin-6, IL-8=interleukin-8, PCT=procalcitonin, sFlt-1=soluble fms-like tyrosine kinase-1, sTNFR1=soluble tumour necrosis factor receptor 1, sTREM-1=soluble triggering receptor expressed on myeloid cells 1, suPAR=soluble urokinase-type plasminogen activator receptor.

Supplementary Figure 4. AUROC of each host biomarker for 7-day mortality among enrolled paediatric and adult patients with febrile illness in southern Mozambique

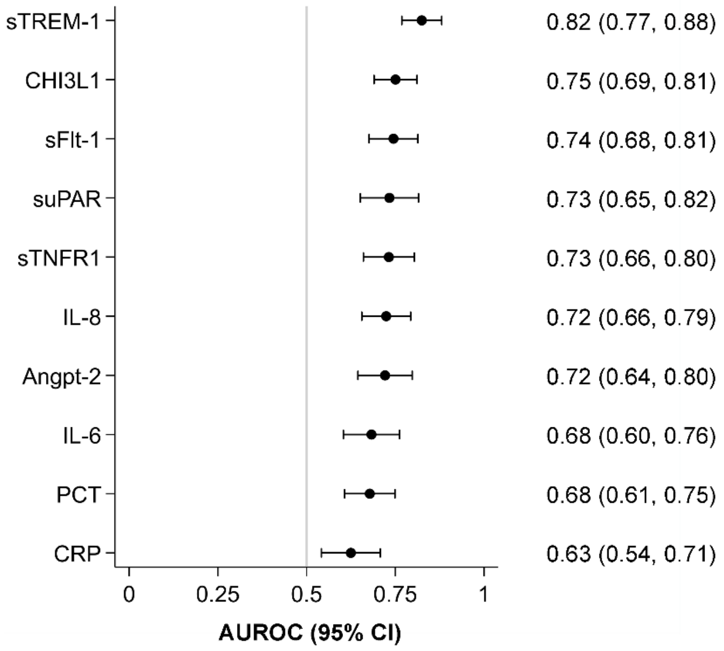

The plot displays the AUROC (dot) with 95% CI (horizontal line) of each biomarker for 7-day mortality. Corresponding numerical values of the AUROCs, with 95% CIs in parentheses, are shown to the right of the plot. The total sample size is N=1,608 (54 deaths). For suPAR, the total sample size is N=1,596 (53 deaths); restricting analyses to this population does not affect the ranking of AUROCs and the AUROC of sTREM-1 remains 0.83 (95% CI: 0.77-0.88).

Abbreviations: Angpt-2=angiopoietin-2, AUROC=area under the receiver operating characteristic curve, CHI3L1=chitinase-3-like protein 1, CI=confidence interval, CRP=C-reactive protein, IL-6=interleukin-6, IL-8=interleukin-8, PCT=procalcitonin, sFlt-1=soluble fms-like tyrosine kinase-1, sTNFR1=soluble tumour necrosis factor receptor 1, sTREM-1=soluble triggering receptor expressed on myeloid cells 1, suPAR=soluble urokinase-type plasminogen activator receptor.

Supplementary Figure 5. sTREM-1 concentrations by vital status at day 28 and HIV status among enrolled paediatric and adult patients with febrile illness in southern Mozambique

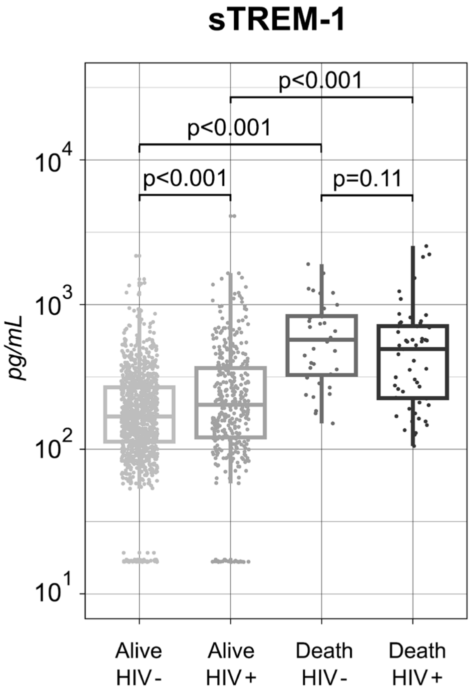

Biomarker concentrations in patients who were HIV-negative and alive at day 28 (N=1,107), HIV-positive and alive at day 28 (N=369), HIV-negative and died by day 28 (N=36), and HIV-positive and died by day 28 (N=55). Boxplots display the median and interquartile range, with whiskers extending to 1.5 times the interquartile range. sTREM-1 concentrations are in pg/mL. p-values were calculated using Mann-Whitney U tests.

Abbreviations: HIV=human immunodeficiency virus, sTREM-1=soluble triggering receptor expressed on myeloid cells 1.

Supplementary Figure 6. Kaplan-Meier survival curves by sTREM-1 categories among different patient subgroups of enrolled paediatric and adult patients with febrile illness in southern Mozambique

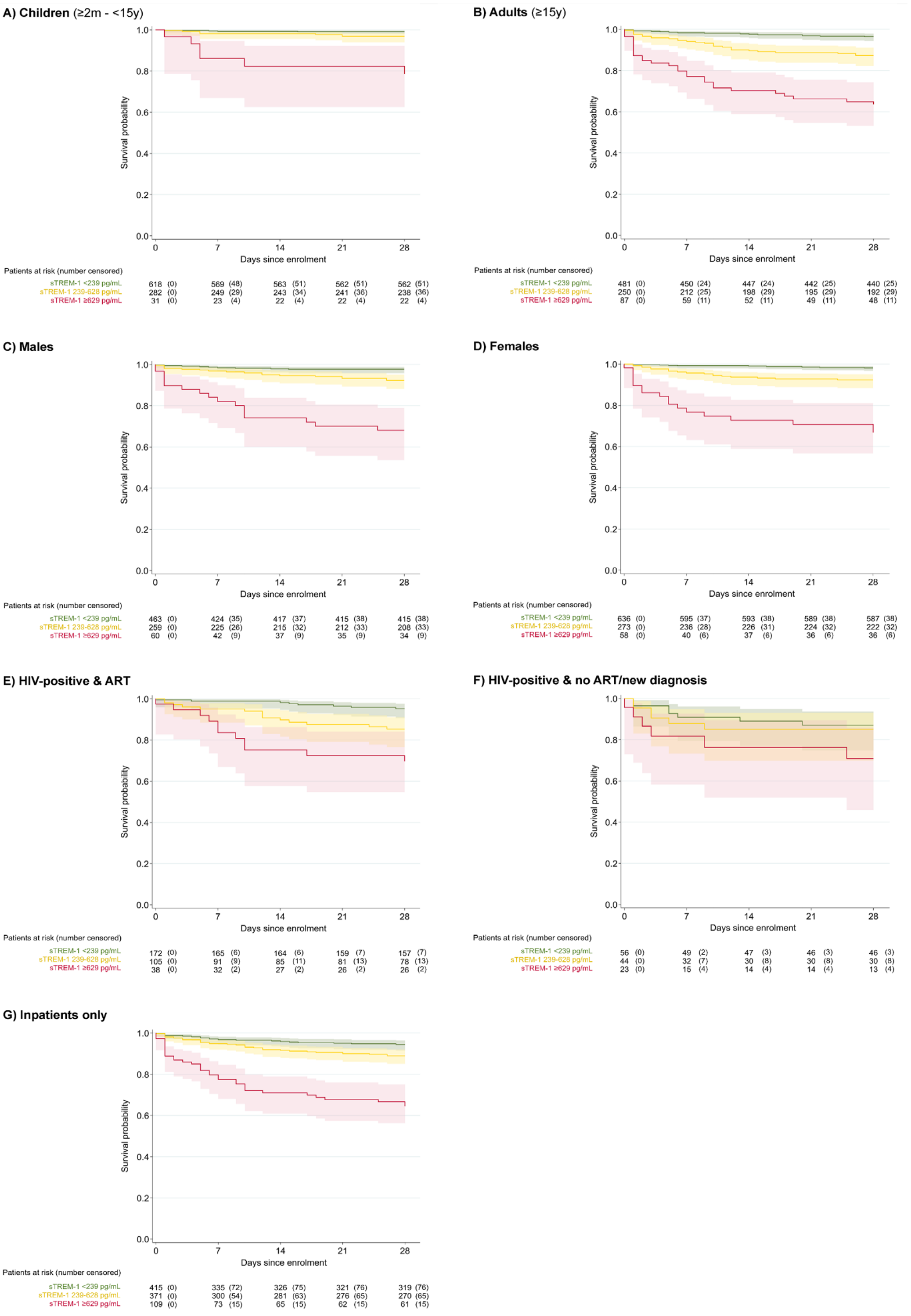

Kaplan-Meier curves with 95% CIs illustrate the survival probability over time by sTREM-1 categories for different patient subgroups (Panels A-G). We applied sTREM-1 cut-offs previously derived with data from hospitalized febrile children in Uganda for mortality prediction [1], stratifying participants into three risk categories based on sTREM-1 levels: low-risk (sTREM-1 <239 pg/mL), intermediate-risk (sTREM-1 239-628 pg/mL), and high-risk (sTREM-1 ≥629 pg/mL). Inpatients with unknown vital status at day 28 were included in the analyses and censored when discharged alive from hospital.

Abbreviations: ART=antiretroviral therapy, CI=confidence interval, HIV=human immunodeficiency virus, sTREM-1=soluble triggering receptor expressed on myeloid cells 1.

Supplementary Figure 7. AUROC of sTREM-1 and lactate for 28-day mortality among enrolled paediatric and adult patients with febrile illness in southern Mozambique

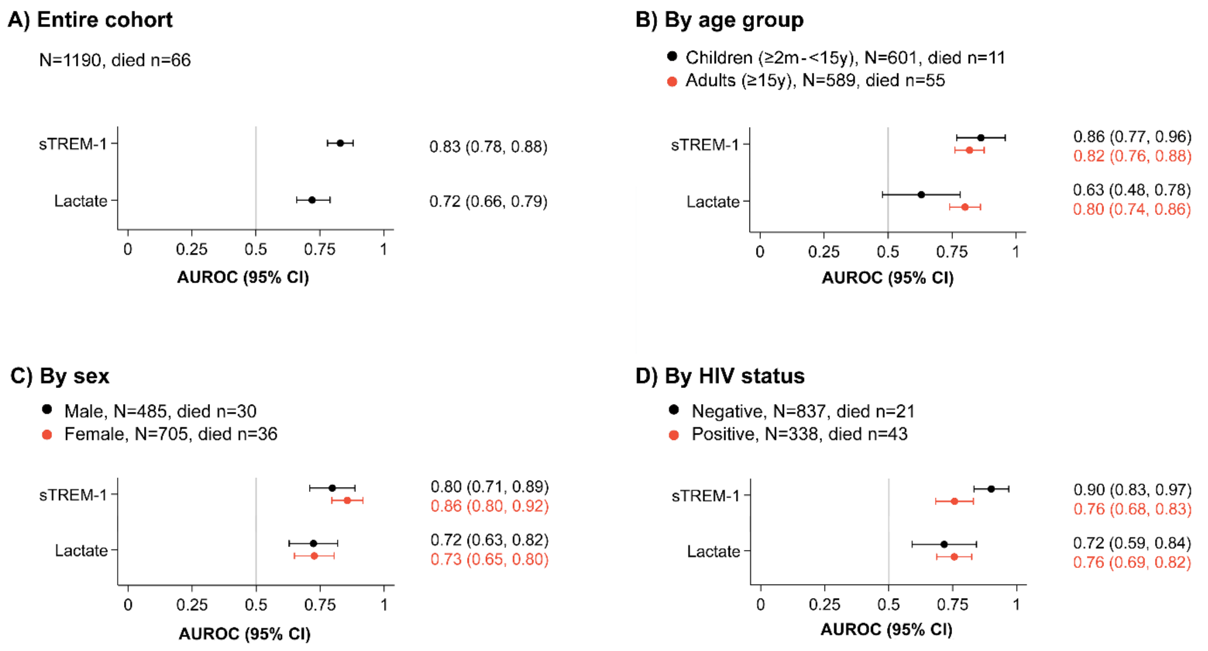

Plots display the AUROC (dot) with 95% CI (horizontal line) of sTREM-1 and lactate for 28-day mortality in the entire cohort (Panel A) and in each patient subgroup according to age group (Panel B), sex (Panel C), and HIV status (Panel D). Corresponding numerical values of the AUROCs, with 95% CIs in parentheses, are shown to the right of each plot.

Abbreviations: CI=confidence interval, AUROC=area under the receiver operating characteristic curve, HIV=human immunodeficiency virus, sTREM-1=soluble triggering receptor expressed on myeloid cells 1.

Supplementary Figure 8. The percentage distribution of each clinical severity score and associated 28-day mortality among enrolled paediatric or adult patients with febrile illness in southern Mozambique

A) ED-PEWS

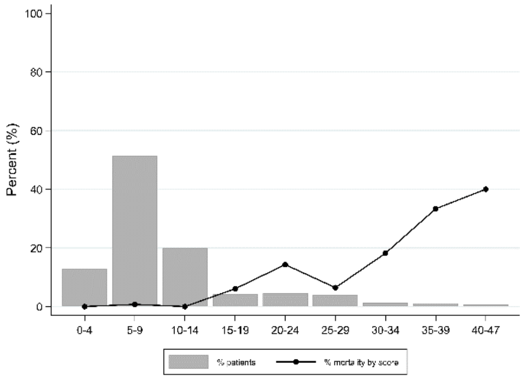

B) LODS

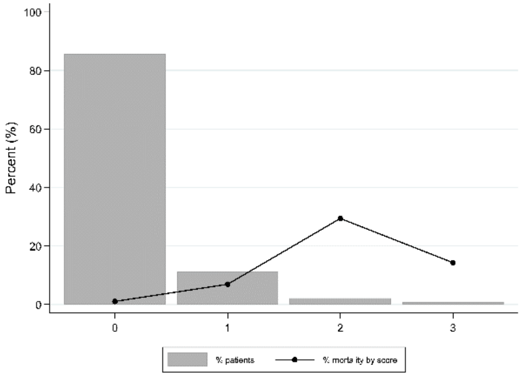

C) LqSOFA

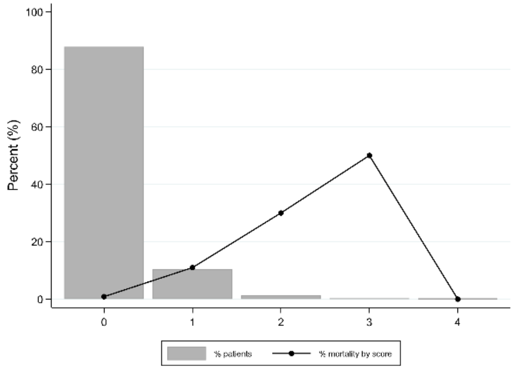

D) MEWS

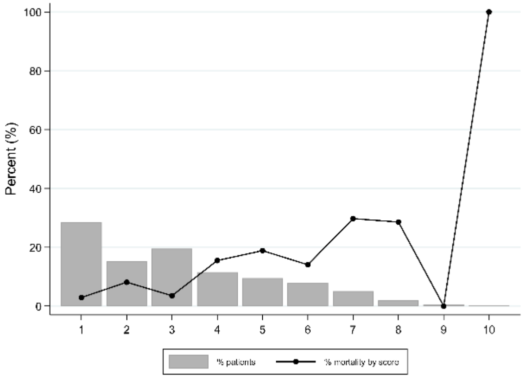

E) qSOFA

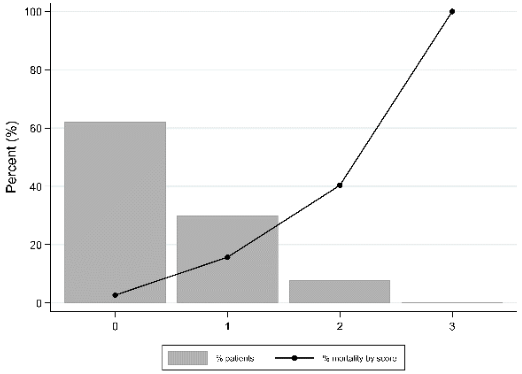

F) UVA

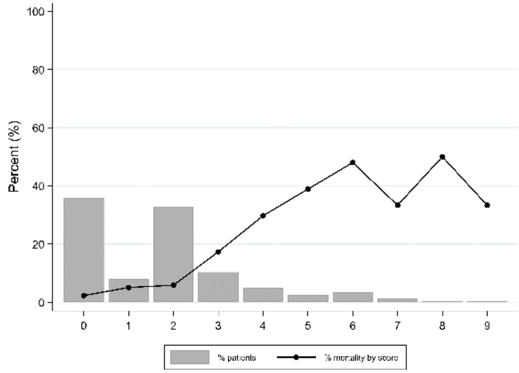

We included all participants with a known vital status at day 28 and clinical data for the calculation of all clinical severity scores. For children (≥2m - <15y) the total sample size is N=784 (Panels A-C), and for adults (≥15y) the total sample size is N=727 (Panels D-F). ED-PEWS scores were grouped in categories to improve visualisation.

Abbreviations: ED-PEWS=Emergency Department Paediatric Early Warning Score, LODS=Lambaréné Organ Dysfunction Score, LqSOFA=Liverpool quick Sequential Organ Failure Assessment, MEWS=Modified Early Warning Score, qSOFA=Quick Sequential (Sepsis-Related) Organ Failure Assessment, UVA=Universal Vital Assessment.

II. SUPPLEMENTARY TABLES

Supplementary Table 1. Percentage and distribution of biomarker values outside the dynamic range

Biomarker	Samples outside the dynamic range, n/N (%)	Number of samples below the dynamic range	Number of samples above the dynamic range
**Luminex:**			
Angpt-2	31/1,955 (1.6%)	2	29
CHI3L1	11/1,955 (0.6%)	11	0
IL-6	210/1,955 (10.7%)	170	40
IL-8	326/1,955 (16.7%)	316	10
PCT	309/1,955 (15.8%)	306	3
sFlt-1	15/1,955 (0.8%)	15	0
sTNFR1	1/1,955 (0.1%)	1	0
sTREM-1	90/1,955 (4.6%)	90	0
**ELISA:**			
CRP	301/1,955 (15.4%)	301	0
suPAR	0/1,939 (0%)	0	0

Abbreviations: Angpt-2=angiopoietin-2, CHI3L1=chitinase-3-like protein 1, CRP=C-reactive protein, ELISA=enzyme-linked immunosorbent assay, IL-6=interleukin-6, IL-8=interleukin-8, PCT=procalcitonin, sFlt-1=soluble fms-like tyrosine kinase-1, sTNFR1=soluble tumour necrosis factor receptor 1, sTREM-1=soluble triggering receptor expressed on myeloid cells 1, suPAR=soluble urokinase-type plasminogen activator receptor.

Supplementary Table 2. Components of the clinical severity scores and their threshold values and points allocated

Emergency Department Paediatric Early Warning Score (ED-PEWS)	Lambaréné Organ Dysfunction Score (LODS)	Liverpool quick Sequential Organ Failure Assessment (LqSOFA) score	Modified Early Warning Score (MEWS)	Quick Sequential (Sepsis-Related) Organ Failure Assessment (qSOFA) score	Universal Vital Assessment (UVA) score
Derived with data from 119,209 children <16 years old attending five diverse emergency departments in Netherland, United Kingdom, Austria, and Portugal. Primary outcome: a three-category reference standard (high, intermediate, low urgency) as a proxy for true patient urgency. Secondary outcomes: hospital admission and intensive care unit admission [2].	Derived with data from 23,890 children ≤15 years old with severe *P. falciparum* malaria hospitalized in one of the six participating African hospitals located in the Gambia, Malawi, Kenya, Ghana, and Gabon. Primary outcome: in-hospital mortality [3].	Derived with data from 1,121 febrile children <16 years old attending the emergency department and requiring a blood test at a specialist paediatric hospital in the United Kingdom. Primary outcome: critical care admission within 48 hours of emergency department attendance. Secondary outcome: sepsis-related mortality [4].	Based on a modified version of the early warning signs score (EWS) [5].	Based on the Third International Consensus Definition for Sepsis and Septic Shock (Sepsis-3) Task Force. It provides simple bedside criteria to identify adult patients with suspected infection who are likely to have poor outcomes [6].	Derived with data from 5,573 patients admitted to hospitals in Gabon, Malawi, Sierra Leone, Tanzania, Uganda, and Zambia. Primary outcome: in-hospital mortality [7].
**Range**: 0-68 points	**Range**: 0-3 points	**Range**: 0-4 points	**Range**: 0-14 points	**Range**: 0-3 points	**Range**: 0-13 points
**Age** (years):<br>0-4: 0 points<br>5-11: 4 points<br>12-16: 6 points<br>**Capillary refill time**:<br>Normal: 0 points<br>Increased: 3 points<br>**Consciousness**:<br>Normal: 0 points<br>Decreased: 14 points<br>**Heart rate** (beats/min):<br><100: 0 points<br>100-139: 3 points<br>140-179: 6 points<br>≥180: 9 points<br>**Oxygen saturation** (%):<br><88: 15 points<br>88-93: 9 points<br>94-97: 4 points<br>≥98: 0 points<br>**Respiratory rate** (breaths/min):<br><30: 0 points<br>30-39: 3 points<br>40-59: 5 points<br>≥60: 9 points<br>**Work of breathing** [a]:<br>Normal: 0 points<br>Increased: 12 points	**Coma** (BCS ≤2):<br>No: 0 points<br>Yes: 1 point<br>**Deep breathing**:<br>No: 0 points<br>Yes: 1 point<br>**Prostration** (not being able to breast-feed, sit, stand, or walk, depending on age):<br>No: 0 points<br>Yes: 1 point	**Capillary refill time** (seconds):<br><3 s: 0 points<br>≥3 s: 1 point<br>**Consciousness** (AVPU scale) [b]:<br>A: 0 points<br>V, P or U: 1 point<br>**Heart rate** (beats/min):<br>≤99th centile Bonafide *et al.* [8] age-specific thresholds: 0 points<br>>99th centile Bonafide *et al.* [8] age-specific thresholds: 1 point<br>**Respiratory rate** (breaths/min):<br>≤99th centile Bonafide *et al.* [8] age-specific thresholds: 0 points<br>>99th centile Bonafide *et al.* [8] age-specific thresholds: 1 point	**Consciousness** (AVPU scale) [b]:<br>A: 0 points<br>V: 1 point<br>P: 2 points<br>U: 3 points<br>**Heart rate** (beats/min):<br>≤40: 2 points<br>41-50: 1 point<br>51-100: 0 points<br>101-110: 1 point<br>111-129: 2 points<br>≥130: 3 points<br>**Respiratory rate** (breaths/min):<br><9: 2 points<br>9-14: 0 points<br>15-20: 1 point<br>21-29: 2 points<br>≥30: 3 points<br>**Systolic blood pressure** (mmHg):<br>≤70: 3 points<br>71-80: 2 points<br>81-100: 1 point<br>101-199: 0 points<br>≥200: 2 points<br>**Temperature** (ºC):<br><35: 2 points<br>35-38.4: 0 points<br>≥38.5: 2 points	**Consciousness** (GCS):<br><15: 1 point<br>15: 0 points<br>**Respiratory rate** (breaths/min):<br><22: 0 points<br>≥22: 1 point<br>**Systolic blood pressure** (mmHg):<br>≤100: 1 point<br>>100: 0 points	**Consciousness** (GCS):<br><15: 4 points<br>15: 0 points<br>**Heart rate** (beats/min):<br><120: 0 points<br>≥120: 1 point<br>**HIV status**:<br>Negative/unknown: 0 points<br>Positive: 2 points<br>**Oxygen saturation** (%):<br><92: 2 points<br>≥92: 0 points<br>**Respiratory rate** (breaths/min):<br><30: 0 points<br>≥30: 1 point<br>**Systolic blood pressure** (mmHg):<br><90: 1 point<br>≥90: 0 points<br>**Temperature** (ºC):<br><36: 2 points<br>≥36: 0 points

[a] In our study, increased work of breathing was defined as difficult breathing, indrawing, deep breathing, or grunting.
[b] In our study, consciousness status was measured using the standard BCS in children and GCS in adults. BCS and GCS were converted to AVPU for LqSOFA and MEWS score calculation.

Abbreviations: AVPU=(A [alert], V [response on verbal stimuli], P [response on pain stimuli], and U [unresponsive]), BCS=Blantyre Coma Scale, GCS=Glasgow Coma Scale, HIV=human immunodeficiency virus.

Supplementary Table 3. Demographic and clinical characteristics of enrolled paediatric patients with febrile illness in southern Mozambique by vital status at day 28

Variable		Children alive at day 28 (N=821)	Children dead by day 28 (N=19)
Sex, n (%)	Female	378 (97.4%)	10 (2.6%)
	Male	443 (98.0%)	9 (2.0%)
Age (years)	Median (IQR)	3.6 (1.8, 6.8)	1.4 (0.7, 4.2)
Age group, n (%)	2 - <12 months	96 (94.1%)	6 (5.9%)
	1 - <5 years	416 (97.9%)	9 (2.1%)
	5 - <15 years	309 (98.7%)	4 (1.3%)
Patient group, n (%)	Inpatient	395 (95.4%)	19 (4.6%)
	Outpatient	426 (100%)	0 (0%)
Site of enrolment, n (%)	Manhiça District Hospital	60 (83.3%)	12 (16.7%)
	Geral José Macamo Hospital	761 (99.1%)	7 (0.9%)
Temperature (°C)	Median (IQR)	38.5 (37.9, 39.1)	38.5 (37.6, 39.0)
Days with fever before enrolment	Median (IQR)	2 (1, 2)	2 (2, 3)
HIV status, n (%) [a]	Positive	46 (86.8%)	7 (13.2%)
	Negative	767 (98.6%)	11 (1.4%)
Malaria status by RDT, n (%) [b]	Positive	187 (100%)	0 (0%)
	Negative	630 (97.4%)	17 (2.6%)
Severe acute malnutrition (WHZ/WLZ <-3), n (%) [c]	Yes	30 (88.2%)	4 (11.8%)
	No	599 (98.8%)	7 (1.2%)
Respiratory rate (breaths/min)	*If 2 - <12 months:* Median (IQR)	38 (34, 50)	60 (48, 70)
	If 1 - <5 years: Median (IQR)	32 (28, 35)	45 (32, 51)
	If 5 - <15 years: Median (IQR)	24 (21, 28)	25 (22, 36)
Systolic blood pressure (mmHg)	*If 2 - <12 months:* Median (IQR)	87 (70, 100)	83 (68, 112)
	If 1 - <5 years: Median (IQR)	89 (80, 100)	90 (69, 105)
	If 5 - <15 years: Median (IQR)	100 (90, 108)	107 (101, 136)
Heart rate (beats/min)	*If 2 - <12 months:* Median (IQR)	132 (114, 144)	153 (130, 180)
	If 1 - <5 years: Median (IQR)	128 (114, 140)	146 (130, 171)
	If 5 - <15 years: Median (IQR)	114 (100, 126)	101 (71, 133)
Capillary refill time (seconds)	Median (IQR)	1 (1, 2)	2 (1, 2)
Work of breathing, n (%) [d]	Increased	85 (88.5%)	11 (11.5%)
	Normal	725 (98.9%)	8 (1.1%)
Deep breathing, n (%)	Yes	25 (92.6%)	2 (7.4%)
	No	790 (97.9%)	17 (2.1%)
Oxygen saturation	Median (IQR)	98 (97, 99)	98 (94, 100)
Altered mental status (BCS <5), n (%)	Yes	33 (76.7%)	10 (23.3%)
	No	760 (98.8%)	9 (1.2%)
Coma (BCS ≤2), n (%)	Yes	11 (68.8%)	5 (31.3%)
	No	782 (98.2%)	14 (1.8%)
Prostration, n (%)	Yes	92 (88.5%)	12 (11.5%)
	No	713 (99.0%)	7 (1.0%)
ED-PEWS	Median (IQR)	8 (7, 11)	24 (16, 35)
LODS	Median (IQR)	0 (0, 0)	1 (0, 2)
LqSOFA	Median (IQR)	0 (0, 0)	1 (0, 1)
Length of hospital stay among inpatients (days)	Median (IQR)	2 (2, 4)	3 (1, 5)
In-hospital outcomes, n (%)	Death	0 (0%)	5 (100%)
	Discharged home	362 (97.7%)	1 (0.3%)
	Absconded	0 (0%)	0 (0%)
	Transferred	15 (55.6%)	12 (44.4%)
Time to death (days)	Median (IQR)	NA	5 (3, 16)

[a] HIV status was considered positive if self-reported by the participant/caregiver or confirmed through HIV point-of-care testing (using antibody-detecting rapid tests) performed for all participants with unknown or negative self-reported HIV status.
[b] Based on antigen-detecting lateral flow malaria RDT, which combines detection of histidine-rich protein 2 and *Plasmodium* lactate dehydrogenase.
[c] Calculated using the WHO Child Growth Standards and the WHO Reference 2007.
[d] Increased work of breathing was defined as difficult breathing, indrawing, deep breathing, or grunting.

Missing data: Days with fever before enrolment, n=15; HIV status, n=9; malaria status by RDT, n=6; severe acute malnutrition, n=200; respiratory rate, n=2; systolic blood pressure, n=89; heart rate, n=4; capillary refill time, n=5; work of breathing, n=11; deep breathing, n=6; oxygen saturation, n=14; altered mental status, n=28; coma, n=28; prostration, n=16; ED-PEWS, n=47; LODS, n=42; LqSOFA, n=34; length of hospital stay among inpatients, n=32 (out of 414 inpatients); in-hospital outcome, n=19 (out of 414 inpatients).

Abbreviations: BCS=Blantyre Coma Scale, ED-PEWS=Emergency Department Paediatric Early Warning Score, HIV=human immunodeficiency virus, IQR=interquartile range, LODS=Lambaréné Organ Dysfunction Score, LqSOFA=Liverpool quick Sequential Organ Failure Assessment, MUAC=mid-upper arm circumference, RDT=rapid diagnostic test, WHZ/WLZ=weight-for-height/length z-score.

Supplementary Table 4. Demographic and clinical characteristics of enrolled adult patients with febrile illness in southern Mozambique by vital status at day 28

Variable		Adults alive at day 28 (N=679)	Adults dead by day 28 (N=74)
Sex, n (%)	Female	465 (92.4%)	38 (7.6%)
	Male	214 (85.6%)	36 (14.4%)
Age (years)	Median (IQR)	34.0 (25.0, 44.0)	42.5 (33.0, 60.0)
Age group, n (%)	15 - <25	166 (97.1%)	5 (2.9%)
	25 - <35	188 (92.2%)	16 (7.8%)
	35 - <45	157 (88.2%)	21 (11.8%)
	45 - <55	73 (91.3%)	7 (8.8%)
	55 - <65	60 (78.9%)	16 (21.1%)
	65+	35 (79.5%)	9 (20.5%)
Patient group, n (%)	Inpatient	253 (77.8%)	72 (22.2%)
	Outpatient	426 (99.5%)	2 (0.5%)
Site of enrolment, n (%)	Manhiça District Hospital	605 (92.2%)	51 (7.8%)
	Geral José Macamo Hospital	74 (76.3%)	23 (23.7%)
Temperature (°C)	Median (IQR)	38.0 (37.7, 38.6)	38.2 (37.9, 38.9)
Days with fever before enrolment	Median (IQR)	3 (2, 4)	4 (2, 7)
HIV status, n (%) [a]	Positive	323 (87.1%)	48 (12.9%)
	Negative	340 (93.2%)	25 (6.8%)
Malaria status by RDT, n (%) [b]	Positive	59 (98.3%)	1 (1.7%)
	Negative	615 (89.5%)	72 (10.5%)
BMI	Median (IQR)	22.5 (20.1, 25.7)	22.1 (18.8, 25.4)
Respiratory rate (breaths/min)	Median (IQR)	19 (18, 20)	22 (20, 28)
Systolic blood pressure (mmHg)	Median (IQR)	117 (106, 130)	115 (96, 131)
Heart rate (beats/min)	Median (IQR)	102 (90, 116)	111 (94, 125)
Capillary refill time (seconds)	Median (IQR)	1 (1, 2)	2 (1, 3)
Oxygen saturation	Median (IQR)	98 (96, 99)	95 (90, 97)
Altered mental status (GCS <15), n (%)	Yes	27 (58.7%)	19 (41.3%)
	No	652 (92.2%)	55 (7.8%)
Coma (GCS ≤8), n (%)	Yes	3 (27.3%)	8 (72.7%)
	No	676 (91.1%)	66 (8.9%)
MEWS	Median (IQR)	3 (1, 4)	5 (3, 6)
qSOFA	Median (IQR)	0 (0, 1)	1 (1, 2)
UVA	Median (IQR)	2 (0, 2)	3 (2, 5)
Length of hospital stay among inpatients (days)	Median (IQR)	3.5 (1, 7)	3 (1, 7)
In-hospital outcomes, n (%)	Death	0 (0%)	34 (100%)
	Discharged home	203 (91.9%)	18 (8.1%)
	Absconded	5 (83.3%)	1 (16.7%)
	Transferred	30 (61.2%)	19 (38.8%)
Time to death (days)	Median (IQR)	NA	6 (1, 12)

[a] HIV status was considered positive if self-reported by the participant/caregiver or confirmed through HIV point-of-care testing (using antibody-detecting rapid tests) performed for all participants with unknown or negative self-reported HIV status.
[b] Based on antigen-detecting lateral flow malaria RDT, which combines detection of histidine-rich protein 2 and *Plasmodium* lactate dehydrogenase.

Missing data: Days with fever before enrolment, n=61; HIV status, n=17; malaria status by RDT, n=6; BMI, n=15; respiratory rate, n=1; systolic blood pressure, n=2; oxygen saturation, n=7; MEWS, n=3; qSOFA, n=3; UVA, n=26; length of hospital stay among inpatients, n=47 (out of 325 inpatients); in-hospital outcome, n=15 (out of 325 inpatients).

Abbreviations: BMI=body mass index, GCS=Glasgow Coma Scale, HIV=human immunodeficiency virus, IQR=interquartile range, MEWS=Modified Early Warning Score, qSOFA=Quick Sequential (Sepsis-Related) Organ Failure Assessment, RDT=rapid diagnostic test, UVA=Universal Vital Assessment.

Supplementary Table 5. Association of host biomarkers with 28-day mortality from univariable logistic regression among enrolled paediatric and adult patients with febrile illness in southern Mozambique

Biomarker	OR (95% CI)	p-value
Angpt-2	2.35 (1.98, 2.79)	<0.001
CHI3L1	1.55 (1.41, 1.71)	<0.001
CRP	1.35 (1.19, 1.52)	<0.001
IL-6	1.30 (1.20, 1.40)	<0.001
IL-8	1.40 (1.28, 1.53)	<0.001
PCT	1.19 (1.12, 1.27)	<0.001
sFlt-1	1.70 (1.42, 2.02)	<0.001
sTNFR1	2.44 (2.02, 2.95)	<0.001
sTREM-1	3.19 (2.57, 3.96)	<0.001
suPAR	2.51 (2.04, 3.10)	<0.001

The total sample size is N=1,593, except for suPAR that is N=1,581. ORs, 95% CIs, and p-values are from univariable logistic regression models. ORs presented indicate the increase in odds of 28-day mortality for every two-fold increase in each biomarker concentration.

Abbreviations: CI=confidence interval, Angpt-2=angiopoietin-2, CHI3L1=chitinase-3-like protein 1, CRP=C-reactive protein, IL-6=interleukin-6, IL-8=interleukin-8, OR=odds ratio, PCT=procalcitonin, sFlt-1=soluble fms-like tyrosine kinase-1, sTNFR1=soluble tumour necrosis factor receptor 1, sTREM-1=soluble triggering receptor expressed on myeloid cells 1, suPAR=soluble urokinase-type plasminogen activator receptor.

Supplementary Table 6. Performance metrics of sTREM-1 cut-offs for predicting 28-day mortality among enrolled paediatric and adult patients with febrile illness in southern Mozambique

Biomarker	Cut-off	Sensitivity (95% CI)	Specificity (95% CI)	LR+ (95% CI)	LR- (95% CI)	PPV (95% CI)	NPV (95% CI)
sTREM-1	<239 pg/mL	77.4 (67.6-85.4)	66.8 (64.4-69.2)	2.33 (2.05-2.66)	0.34 (0.23-0.49)	12.6 (10.0-15.6)	97.9 (96.9-98.7)
	≥629 pg/mL	37.6 (27.8-48.3)	95.5 (94.3-96.5)	8.30 (5.85-11.78)	0.65 (0.56-0.77)	34.0 (24.9-44.0)	96.1 (95.0-97.0)

Abbreviations: CI=confidence interval, LR+=positive likelihood ratio, LR-=negative likelihood ratio, NPV=negative predictive value, PPV=positive predictive value, sTREM-1=soluble triggering receptor expressed on myeloid cells 1.

Supplementary Table 7. Association of sTREM-1 and clinical severity scores with 28-day mortality from univariable and multivariable logistic regression among enrolled paediatric or adult patients with febrile illness in southern Mozambique

**CHILDREN (≥2m - <15y), N=784**		
**Variable**	**OR (95% CI)**	**p-value**
sTREM-1	4.67 (2.76, 7.90)	<0.001
ED-PEWS	1.15 (1.11, 1.20)	<0.001
LODS	3.85 (2.41, 6.16)	<0.001
LqSOFA	5.20 (2.90, 9.31)	<0.001
sTREM-1 adjusted for ED-PEWS	2.65 (1.48, 4.74)	0.0011
sTREM-1 adjusted for LODS	3.29 (1.88, 5.75)	<0.001
sTREM-1 adjusted for LqSOFA	3.35 (1.93, 5.82)	<0.001
**ADULTS (≥15y), N=727**		
**Variable**	**OR (95% CI)**	**p-value**
sTREM-1	2.48 (1.97, 3.13)	<0.001
MEWS	1.44 (1.29, 1.62)	<0.001
qSOFA	5.08 (3.52, 7.32)	<0.001
UVA	1.72 (1.51, 1.96)	<0.001
sTREM-1 adjusted for MEWS	2.23 (1.76, 2.82)	<0.001
sTREM-1 adjusted for qSOFA	2.02 (1.58, 2.58)	<0.001
sTREM-1 adjusted for UVA	2.10 (1.64, 2.68)	<0.001

We included all participants with a known vital status at day 28 and clinical data for the calculation of all clinical severity scores. ORs, 95% CIs, and p-values are from univariable or multivariable logistic regression models. For sTREM-1, ORs presented indicate the increase in odds of 28-day mortality for every two-fold increase in sTREM-1 concentration. For clinical severity scores, ORs presented indicate the increase in odds of 28-day mortality for each one-unit increase in the score.

Abbreviations: CI=confidence interval, ED-PEWS=Emergency Department Paediatric Early Warning Score, LODS=Lambaréné Organ Dysfunction Score, LqSOFA=Liverpool quick Sequential Organ Failure Assessment, MEWS=Modified Early Warning Score, OR=odds ratio, qSOFA=Quick Sequential (Sepsis-Related) Organ Failure Assessment, sTREM-1=soluble triggering receptor expressed on myeloid cells 1, UVA=Universal Vital Assessment.

Supplementary Table 8. AUROC of each host biomarker for predicting adverse outcomes other than mortality among enrolled paediatric and adult outpatients with febrile illness in southern Mozambique

	Seeking further care for the same illness by the follow-up visit in outpatients, AUROC + 95% CI (N=838)	Subsequent hospitalisation for the same illness by the follow-up visit in outpatients, AUROC + 95% CI (N=838)
Angpt-2	0.55 (0.47, 0.62)	0.64 (0.42, 0.85)
CHI3L1	0.55 (0.49, 0.62)	0.65 (0.48, 0.81)
CRP	0.57 (0.49, 0.65)	0.73 (0.63, 0.83)
IL-6	0.56 (0.48, 0.64)	0.74 (0.65, 0.84)
IL-8	0.53 (0.46, 0.60)	0.66 (0.48, 0.85)
PCT	0.61 (0.53, 0.68)	0.80 (0.68, 0.92)
sFlt-1	0.58 (0.50, 0.65)	0.59 (0.41, 0.77)
sTNFR1	0.60 (0.53, 0.67)	0.79 (0.68, 0.90)
sTREM-1	0.62 (0.55, 0.69)	0.73 (0.60, 0.87)
suPAR	0.61 (0.54, 0.69)	0.79 (0.62, 0.96)

Missing data: All included patients had complete biomarker data.

Abbreviations: Angpt-2=angiopoietin-2, CHI3L1=chitinase-3-like protein 1, CRP=C-reactive protein, IL-6=interleukin-6, IL-8=interleukin-8, PCT=procalcitonin, sFlt-1=soluble fms-like tyrosine kinase-1, sTNFR1=soluble tumour necrosis factor receptor 1, sTREM-1=soluble triggering receptor expressed on myeloid cells 1, suPAR=soluble urokinase-type plasminogen activator receptor.

III. SUPPLEMENTARY REFERENCES

1 Leligdowicz A, Conroy AL, Hawkes M, *et al.* Risk-stratification of febrile African children at risk of sepsis using sTREM-1 as basis for a rapid triage test. *Nat Commun* 2021; **12**: 6832.

2 Zachariasse JM, Nieboer D, Maconochie IK, *et al.* Development and validation of a Paediatric Early Warning Score for use in the emergency department: a multicentre study. *The Lancet Child & Adolescent Health* 2020; **4**: 583–91.

3 Helbok R, Kendjo E, Issifou S, *et al.* The Lambaréné Organ Dysfunction Score (LODS) Is a Simple Clinical Predictor of Fatal Malaria in African Children. *J Infect Dis* 2009; **200**: 1834–41.

4 Romaine ST, Potter J, Khanijau A, *et al.* Accuracy of a Modified qSOFA Score for Predicting Critical Care Admission in Febrile Children. *Pediatrics* 2020; **146**: e20200782.

5 Subbe CP. Validation of a modified Early Warning Score in medical admissions. *QJM* 2001; **94**: 521–6.

6 Singer M, Deutschman CS, Seymour CW, *et al.* The Third International Consensus Definitions for Sepsis and Septic Shock (Sepsis-3). *JAMA* 2016; **315**: 801–10.

7 Moore CC, Hazard R, Saulters KJ, *et al.* Derivation and validation of a universal vital assessment (UVA) score: a tool for predicting mortality in adult hospitalised patients in sub-Saharan Africa. *BMJ Glob Health* 2017; **2**: e000344.

8 Bonafide CP, Brady PW, Keren R, *et al.* Development of heart and respiratory rate percentile curves for hospitalized children. *Pediatrics* 2013; **131**: e1150–7.
